# Crystal structures of five halido gold complexes involving piperidine or pyrrolidine as ligands or (protonated) as cations^1^

**DOI:** 10.1107/S205698902300854X

**Published:** 2023-10-10

**Authors:** Cindy Döring, Peter G. Jones

**Affiliations:** a Institut für Anorganische und Analytische Chemie, Technische Universität Braunschweig, Hagenring 30, D-38106 Braunschweig, Germany; Universität Greifswald, Germany

**Keywords:** crystal structure, gold, piperidine, pyrrolidine, secondary inter­actions

## Abstract

In bromido­(pyrrolidine-κ*N*)gold(I) bis­(pyrrolidine-κ*N*)gold(I) bromide, [AuBr(pyr)]·[Au(pyr)_2_]Br (pyr = pyrrolidine, C_4_H_9_N), **2**, alternating [AuBr(pyr)] mol­ecules and [Au(pyr)_2_]^+^ cations are connected by aurophilic contacts to form infinite chains of residues parallel to the *b* axis. The chains are cross-linked by three N—H⋯Br^−^ hydrogen bonds and an Au⋯Br contact to form a layer structure parallel to the *ab* plane. Tri­chlorido­(piperidine-κ*N*)gold(III), [AuCl_3_(pip)] (pip = piperidine, C_5_H_11_N), **3**, consists of mol­ecules with the expected square-planar coordination at the gold atom, which are connected by an N—H⋯Cl hydrogen bond and an Au⋯Cl contact to form a layer structure parallel to the *ac* plane. The structures of bis­(piperidinium) tetra­chlorido­aurate(III) chloride, (pipH)_2_[AuCl_4_]Cl, **4**, and bis­(pyrrolidinium) tetra­bromido­aurate(III) bromide, (pyrH)_2_[AuBr_4_]Br, **6**, are closely related but not isotypic. Compound **6** crystallizes in space group *Ibam*; the Au and two Br atoms of the anion lie in the mirror plane *x*, *y*, 0, whereas the bromide ions occupy special positions 0, 0.5, 0 and 0, 0.5, 0.25, with site symmetry 2/*m*. The NH_2_ group forms a hydrogen bond to one bromide ion, and also a three-centre hydrogen bond to the other bromide atom and to a metal-bonded Br atom. The packing involves chains of hydrogen-bonded pyrrolidinium and bromide ions parallel to the *c* axis, combined with a layer structure of [AuBr_4_]^−^ and bromide anions, parallel to the *ab* plane and involving Au⋯Br and Br⋯Br contacts. Compound **4**, however, crystallizes pseudosymmetrically in space group *Iba*2; two chlorine atoms of the anion lie on the twofold axis 0.5, 0.5, *z*, and there are two independent cations. The packing is closely similar to that of **6**, but there are no N—H⋯Cl hydrogen bonds to metal-bonded chlorines. The contact distances Au⋯Cl are appreciably longer than their Au⋯Br counterparts in **6**, whereas the Cl⋯Cl contact is much shorter than Br⋯Br in **6**. Tri­bromido­(piperidine-κ*N*)gold(III) crystallizes as its di­chloro­methane solvate, [AuBr_3_(pip)]·CH_2_Cl_2_, **7**. It too displays a square-planar coordination at the gold atom. The packing involves hydrogen bonds N—H⋯Br, stacking of neighbouring AuBr_3_ units by Au⋯Br contacts, and a short Br⋯Br contact; these combine to form a layer structure parallel to the *ac* plane.

## Abstract 2

The structures of five gold complexes involving piperidine or pyrrolidine, either as neutral ligands or protonated counter-cations, are presented.

## 1. Chemical context

**Figure 1:**
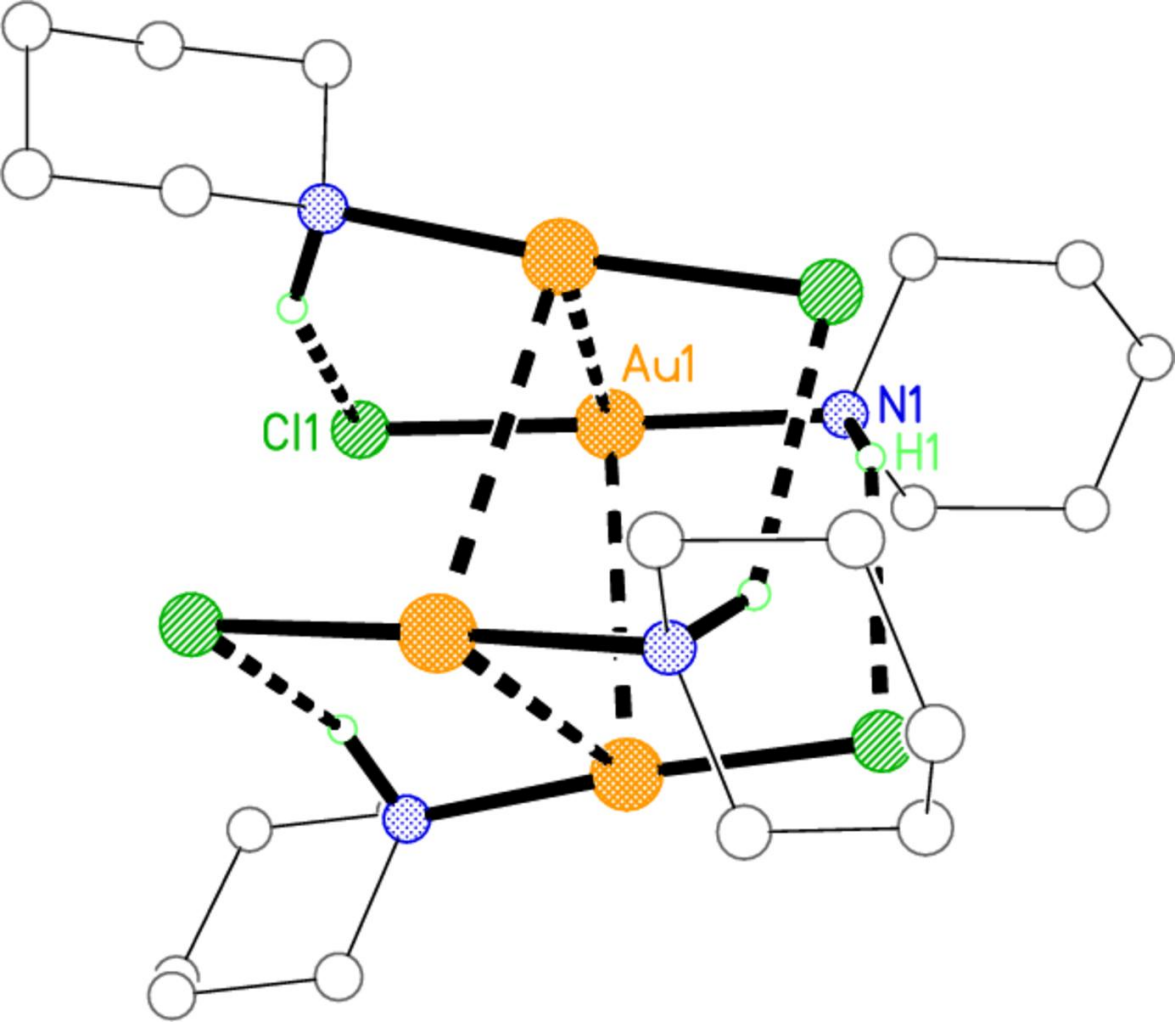
Structure of the tetra­meric unit of [AuCl(pip)] (Guy *et al.*, 1977 ▸), which displays crystallographic 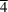 symmetry. Radii are arbitrary. Dashed lines indicate short Au⋯Au contacts or H⋯Cl hydrogen bonds. Throughout this paper, hydrogen atoms of the CH_2_ groups are omitted from the packing diagrams for clarity.

According to the well-known classification of metal ions and ligands introduced by Pearson (1963 ▸), gold(I), and to a lesser extent gold(III), are regarded as archetypal ‘soft’ metal centres and, as such, would be expected to form stable complexes with soft ligands (typically with donor atoms such as sulfur and phospho­rus) rather than hard ligands (with *e.g*. nitro­gen or oxygen donors). As a general trend this is true, but even gold(I) nevertheless forms a wide variety of complexes with nitro­gen ligands such as amines (in which we include aza-aromatics). We have studied these extensively, particularly with regard to their structural aspects, in the series of papers ‘Gold complexes with amine ligands’, of which this forms the latest part; part 1 appeared in 1997 (Jones & Ahrens, 1997 ▸) and the previous part (part 11) in 2018 (Döring & Jones, 2018*b* ▸). However, our first (unnumbered) paper on the subject appeared almost half a century ago (Guy *et al.*, 1977 ▸), and concerned complexes of the cyclic secondary amine piperidine (henceforth ‘pip’ in formulae). Crystals of [AuCl(pip)] were obtained in small qu­anti­ties in an attempt to crystallize the complex [Au(pip)_2_]Cl; the structure was determined, and consisted, predictably, of mol­ecules with linear coordination at gold. Quite unpredictable at the time was the fact that the mol­ecules associated to form tetra­mers (Fig. 1 ▸) based on an approximately square quadrilateral of gold atoms with short Au⋯Au contacts of 3.301 (5) Å, Au⋯Au⋯Au angles of 88.3° and deviations from the plane of ±0.29 Å. A literature survey ‘X-Ray structural investigations of gold compounds’ by one of us (Jones, 1981 ▸) presented numerous examples of structures with such Au^I^⋯Au^I^ contacts, later termed ‘aurophilic contacts’ by Schmidbaur, who published extensively on the subject (see *e.g*. Schmidbaur & Schier, 2008 ▸, 2012 ▸).

Two additional features of the [AuCl(pip)] structure remained unnoticed (or at least were not commented on) at the time. First, the structure contains N—H⋯Cl hydrogen bonds between adjacent mol­ecules of the tetra­mer, with H⋯Cl 2.57 and N—H⋯Cl 136° (and a possible weaker branch of a three-centre hydrogen bond, with H⋯Cl 2.91 and N—H⋯Cl 127°); hydrogen bonds involving halides bonded to metals are now an established concept, thanks to extensive research by Brammer and others (see *e.g.* Brammer, 2003 ▸). Secondly, the substituents *Z* at a piperidine ring may adopt an axial or an equatorial position, with C—C—N—*Z* torsion angles of approximately 180°, or an axial position, with values of approximately 60°. Because the equatorial positions are sterically more favourable, these would tend to be occupied preferentially, and this is indeed the case for [AuCl(pip)], with C—C—N—Au torsion angles of ±176°. Clearly a modern redetermination of the structure of [AuCl(pip)] would be worthwhile, for instance to determine directly the positions of the NH hydrogen atom (which had been positioned geometrically, as was normal at the time, rather than directly located and refined). However, despite repeated attempts, we have never again succeeded in synthesizing or crystallizing the complex.

Much later, we succeeded in determining the structure of [Au(pip)_2_]Cl (Ahrens *et al.*, 1999 ▸), which consists of inversion-symmetric dimers with NH⋯Cl^−^⋯HN linkages. The Au⋯Au distance of 4.085 (2) Å within the dimers is too long to be considered an inter­action.

Investigations using the closely related heterocycle pyrrolidine (henceforth ‘pyr’ in formulae) established the structures of [Au(pyr)_2_]Cl [as its di­chloro­methane (2/3)-solvate; Ahrens *et al.*, 1999 ▸] and 2[AuCl(pyr)]·[Au(pyr)_2_]Cl (≡Au_3_Cl_3_(pyr)_4_; Jones & Ahrens, 1997 ▸). The former consists of trimeric units based on a linear chain of three gold atoms linked by aurophilic inter­actions. The trimers are further linked by N—H⋯Cl^−^ hydrogen bonds to form a ribbon structure, which contains infinite undulating chains of [AuCl(pyr)] and [Au(pyr)_2_]^+^ residues linked by aurophilic inter­actions, with N—H⋯Cl^−^ hydrogen bonds acting as struts across the bends in the chain.

**Figure 2:**
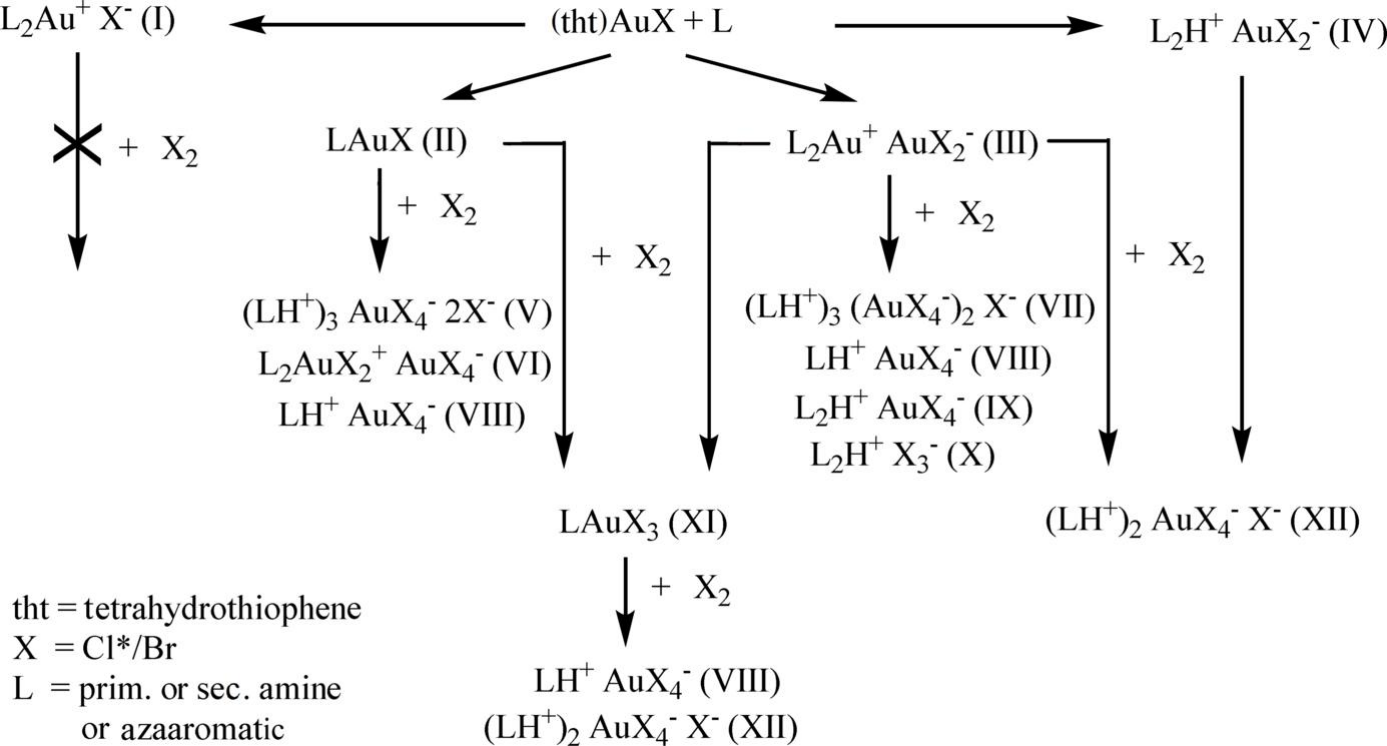
Schematic summary of various structure types for derivatives of amines with gold halides. *X_2_ in the case of chlorine refers to the chlorinating agent PhICl_2_ rather than elemental chlorine, the use of which has practical difficulties.

Our initial studies involved derivatives of gold(I) chloride, because the easiest access to the amine complexes is the reaction of the amine *L* (generally as a neat liquid, because many of the products are only stable in the presence of excess amine) with AuCl complexes containing easily displaced ligands such as tetra­hydro­thio­phene (tht) or dimethyl sulfide. However, the products are not easily predictable and can be of various types such as [AuCl*L*], [Au*L*_2_]Cl or [Au*L*_2_][AuCl_2_] (see Fig. 2 ▸, which presents an overview of various product types that have been established during our investigations) or in rare cases a mixture such as Au_3_Cl_3_ *L*_4_, as mentioned above for pyrrolidine.

**Figure 3:**
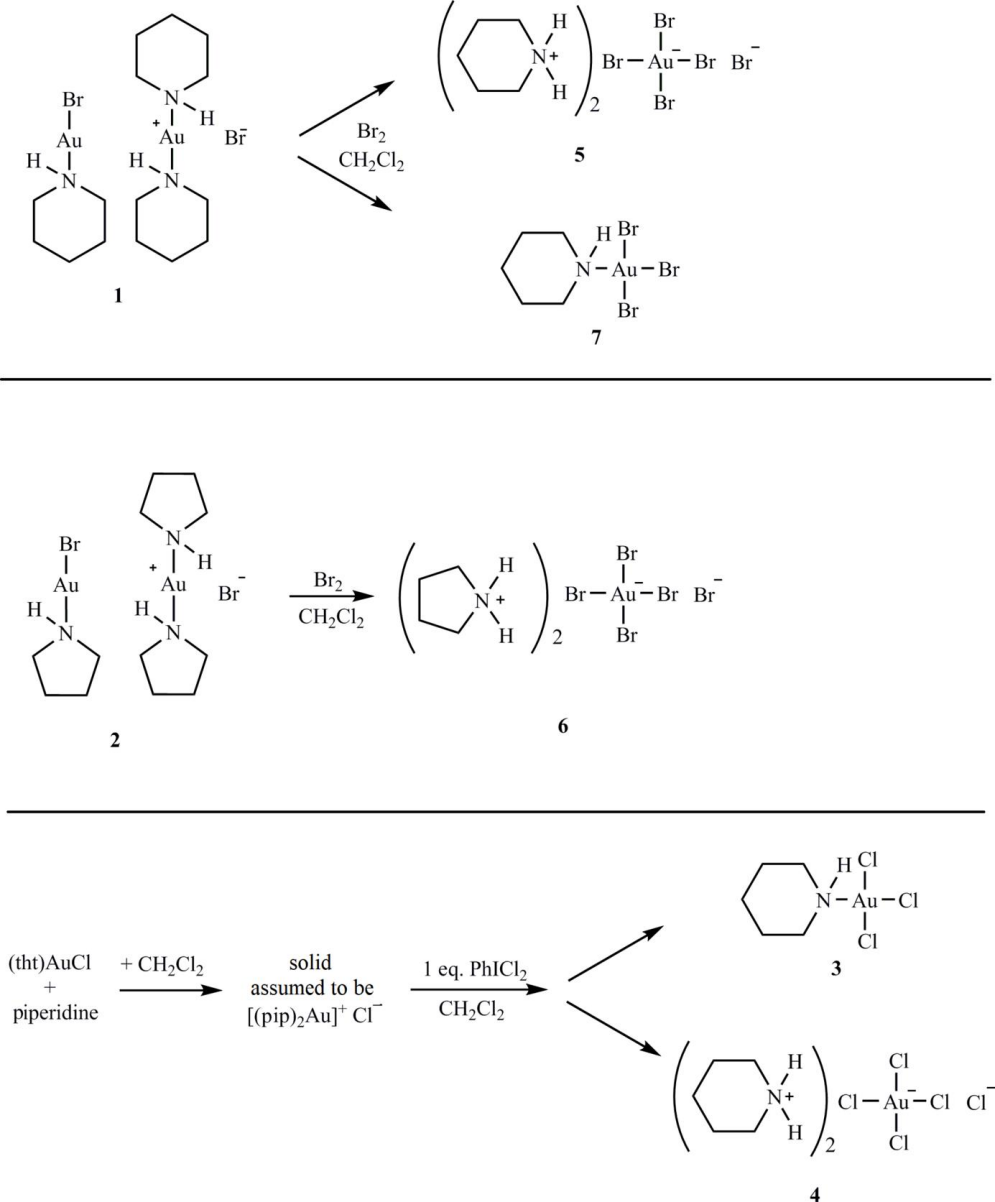
Preparative pathways to the compounds discussed in this paper.

We have since extended the studies to bromide, cyanide (Döring & Jones, 2013 ▸) and thio­cyanate (Strey *et al.*, 2018 ▸) complexes of gold(I). A further extension has been the attempt to oxidize the gold(I) derivatives to gold(III) analogues. Our studies have however unfortunately shown that these reactions (typically using the oxidising agents PhICl_2_ or elemental bromine) often lead to intra­ctable mixtures of products, and that some reactions are extremely sensitive to traces of H^+^ (arising perhaps from adventitious water or by reactions with the solvent), leading to salts of the protonated amine with [Au*X*_4_]^−^ and *X* ^−^ anions. Crystallization processes tend to be slow and can lead to decomposition products rather than the intended [Au*X*_3_ *L*] (*L* = amine, *X* = Cl or Br). Nevertheless, the structures thus obtained display some inter­esting features, which compensate to some extent for the disappointing lack of synthetic efficiency. Here we present the structures of the Au^I^ derivative [AuBr(pyr)]·[Au(pyr)_2_]Br (**2**) (≡Au_2_Br_2_(pyr)_3_), together with the Au^III^ derivatives [AuCl_3_(pip)] (**3**), (pipH)_2_Cl[AuCl_4_] (**4**), the closely related pyrrolidine complex (pyrH)_2_Br[AuBr_4_] (**6**), and [AuBr_3_(pip)] as its di­chloro­methane solvate **7** (see below). For the sake of completeness, we also make brief reference to the structures of [AuBr(pip)]·[Au(pip)_2_]Br (**1**) and (pipH)_2_Br[AuBr_4_] (**5**), which were however of poor quality. The preparative pathways to these compounds are summarized in Fig. 3 ▸. 
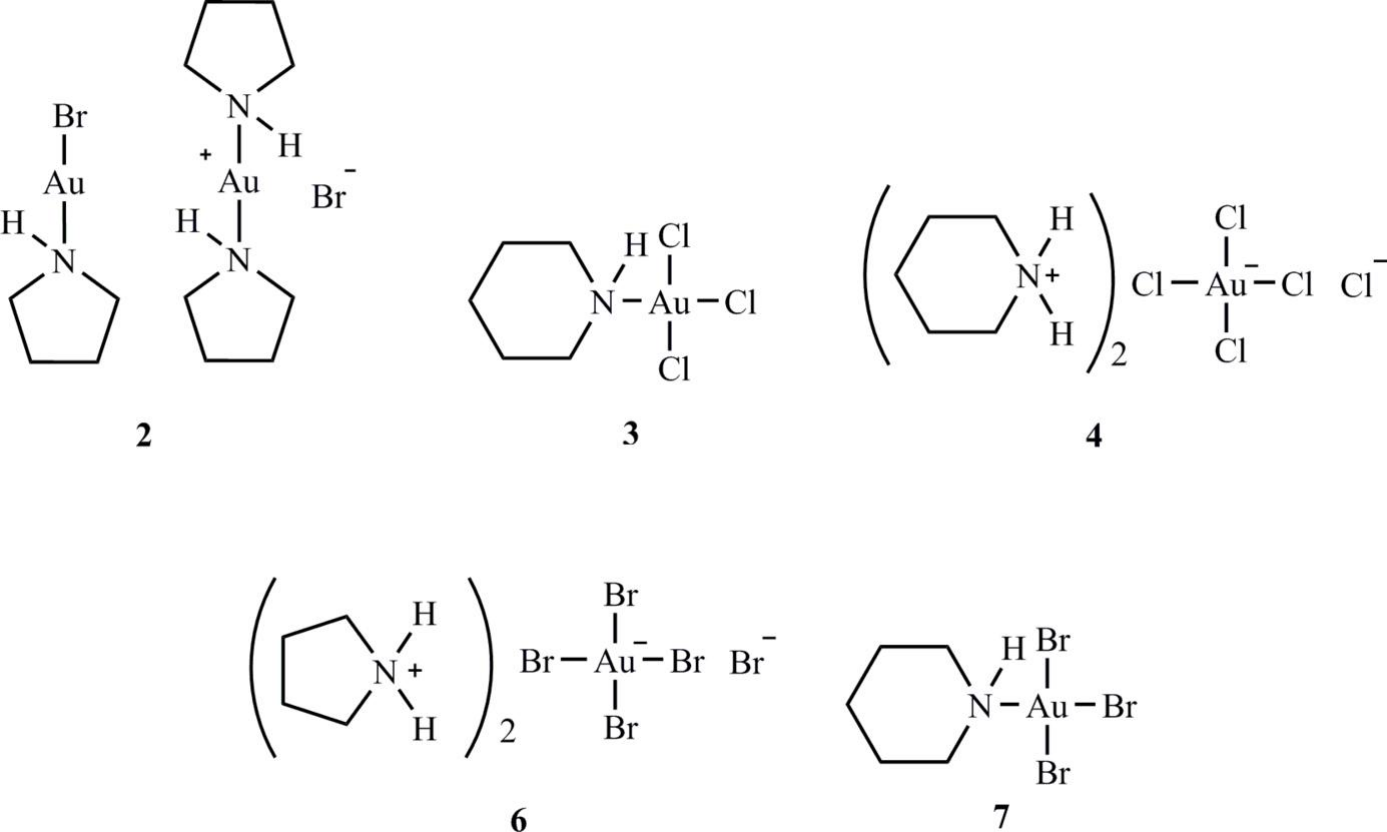


## 2. Structural commentary

At the outset it should be noted that, for the structures **2**, **4** and **6**, which contain more than one residue in the asymmetric unit, the distinction between the categories *Structural commentary* (which generally refers to the asymmetric unit) and *Supra­molecular features* becomes blurred.

**Figure 4:**
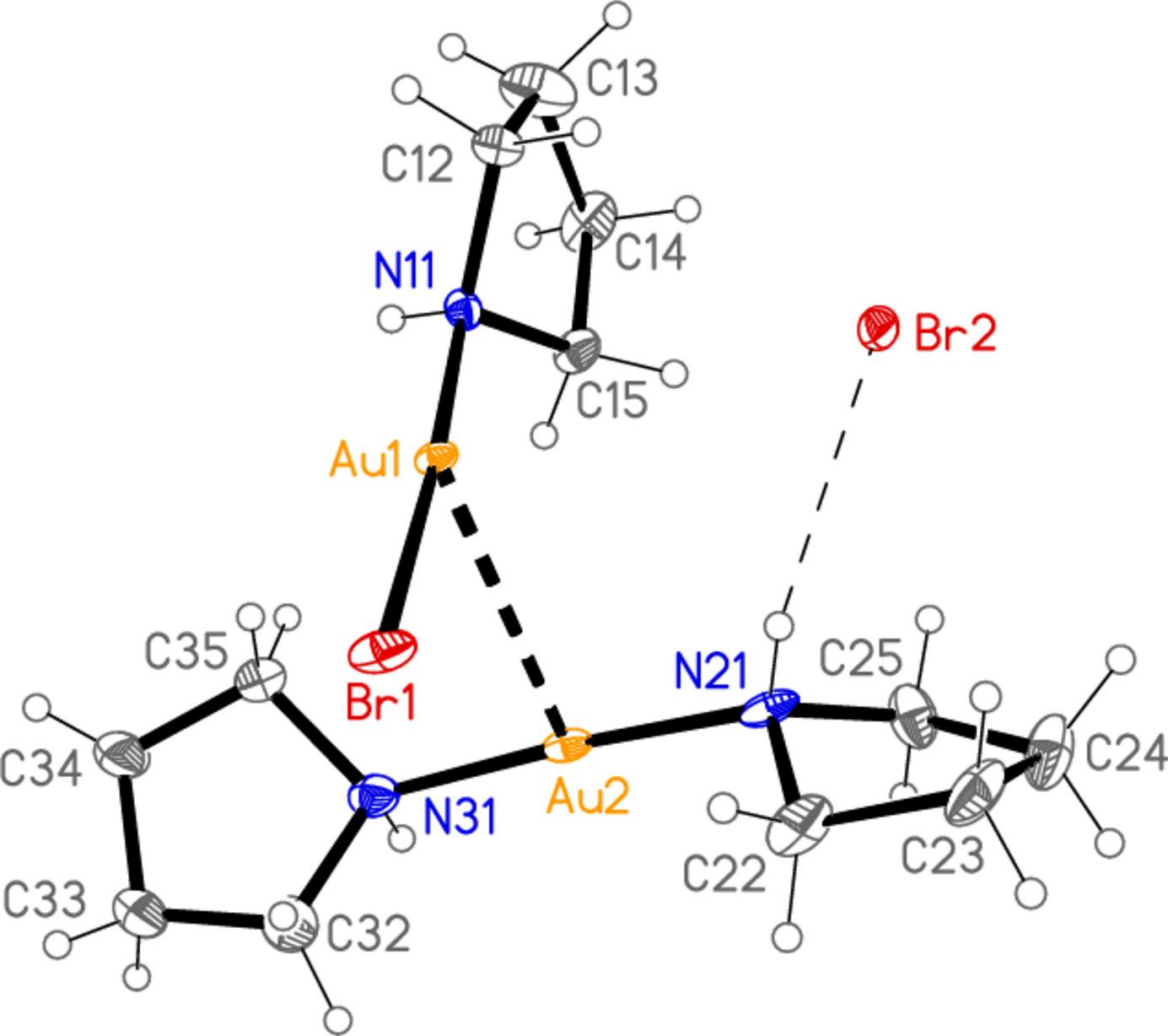
The asymmetric unit of compound **2** with ellipsoids at the 30% probability level. The minor disorder component is not shown. The dashed lines represent the Au⋯Au contact and the hydrogen bond.

The formula unit of compound **2** is shown in Fig. 4 ▸. For reasons discussed below, the structure is only of moderate quality, but it provides important information on this structure type, which differs in stoichiometry from the chlorido complexes in our previous publications. The compound is formally a 1:1 adduct of [AuBr(pyr)] and [Au(pyr)_2_]Br (types II and I respectively according to our arbitrary classification). The neutral mol­ecule and the cation display bond lengths and angles that may be regarded as normal (Table 1 ▸). The Au1—N11 bond *trans* to Br1 is significantly longer than the Au2—N bonds of the cation. The six absolute torsion angles Au—N—C—C all lie in the range 157–173°. The gold atoms are connected by a short aurophilic contact Au1⋯Au2 3.1478 (6) Å; the residues lie with the coordination axes at the gold atoms almost perpendicular to each other, so that the torsion angles about the Au1⋯Au2 contact are all roughly 90°. The NH group at N21 makes a classical hydrogen bond to the bromide anion Br2 (Table 2 ▸). The unsatisfactory structure of compound **1** is effectively isotypic to **2**; in particular, the absolute torsion angles Au—N—C—C all lie in the narrow range 176–180°, so that the gold atoms lie equatorially with respect to the piperidine rings.

**Figure 5:**
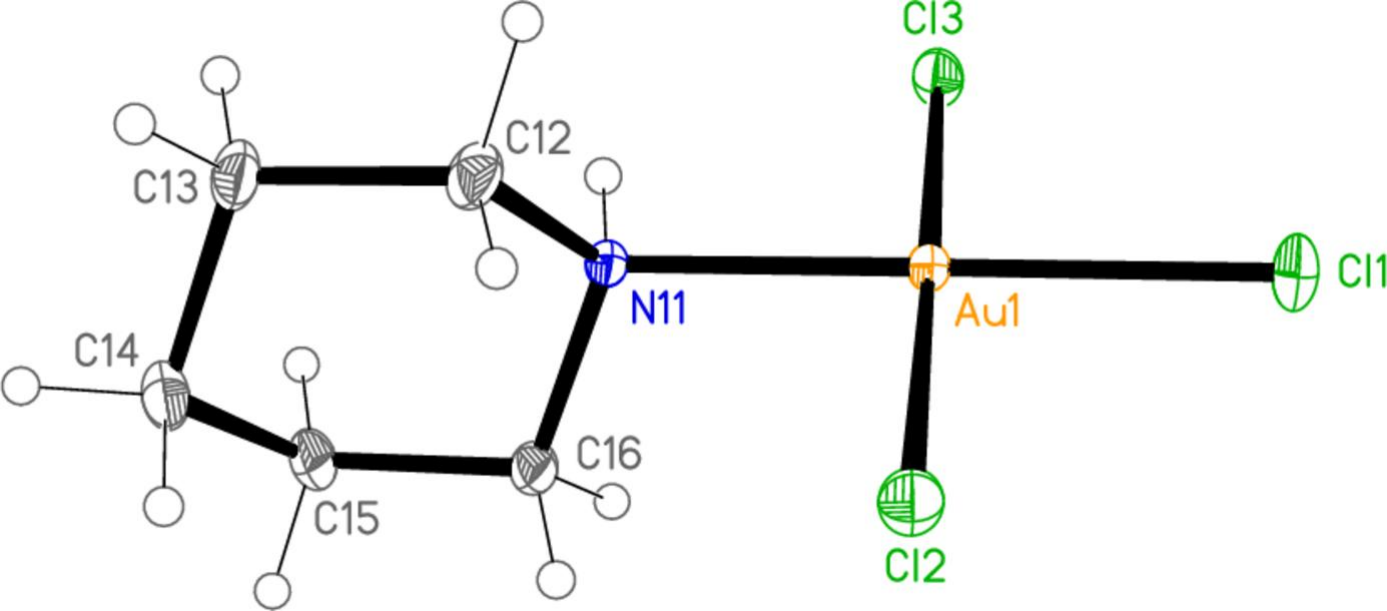
The asymmetric unit of compound **3** with ellipsoids at the 50% probability level.

The mol­ecular structure of compound **3** (type XI) is shown in Fig. 5 ▸. The coordination geometry at the gold atom is, as expected, square planar; the r.m.s. deviation of Au1 and its bonded atoms from the plane that they define is only 0.014 Å. Bond lengths and angles (Table 3 ▸) are normal; the Au1—Cl1 bond length (*trans* to N11) is not markedly different from the other Au—Cl bond lengths. The Au—N—C—C torsion angles are close to ±180°, so that the AuCl_3_ moiety lies in an equatorial position with respect to the piperidine ring. The short intra­molecular contact H01⋯Cl3 of 2.57 (3) Å might represent a hydrogen bond (Table 4 ▸), although the angle is necessarily far from linear at 114 (2)°; the synperiplanar disposition of H01, N11, Au1 and Cl3, torsion angle −8 (2)°, would be consistent with this inter­pretation.

In a parallel attempt to obtain compound **3**, the piperidinium salt (pipH)_2_Cl[AuCl_4_] (**4**; type XII) was obtained. Analogous attempts to obtain [AuBr_3_(pip)] (**7**; see below) and [AuBr_3_(pyr)] led to (pipH)_2_Br[AuBr_4_] (**5**) and (pyrH)_2_Br[AuBr_4_] (**6**). Compounds **4**, **5** and **6** all crystallized with closely similar cells and with systematic absences corresponding to space groups *Ibam* or *Iba*2. However, the structures of **4** and **5** proved to be pseudosymmetric, and **5** could not be successfully refined, so we discuss the structure of **6** first and then the less straightforward structure of **4**.

**Figure 6:**
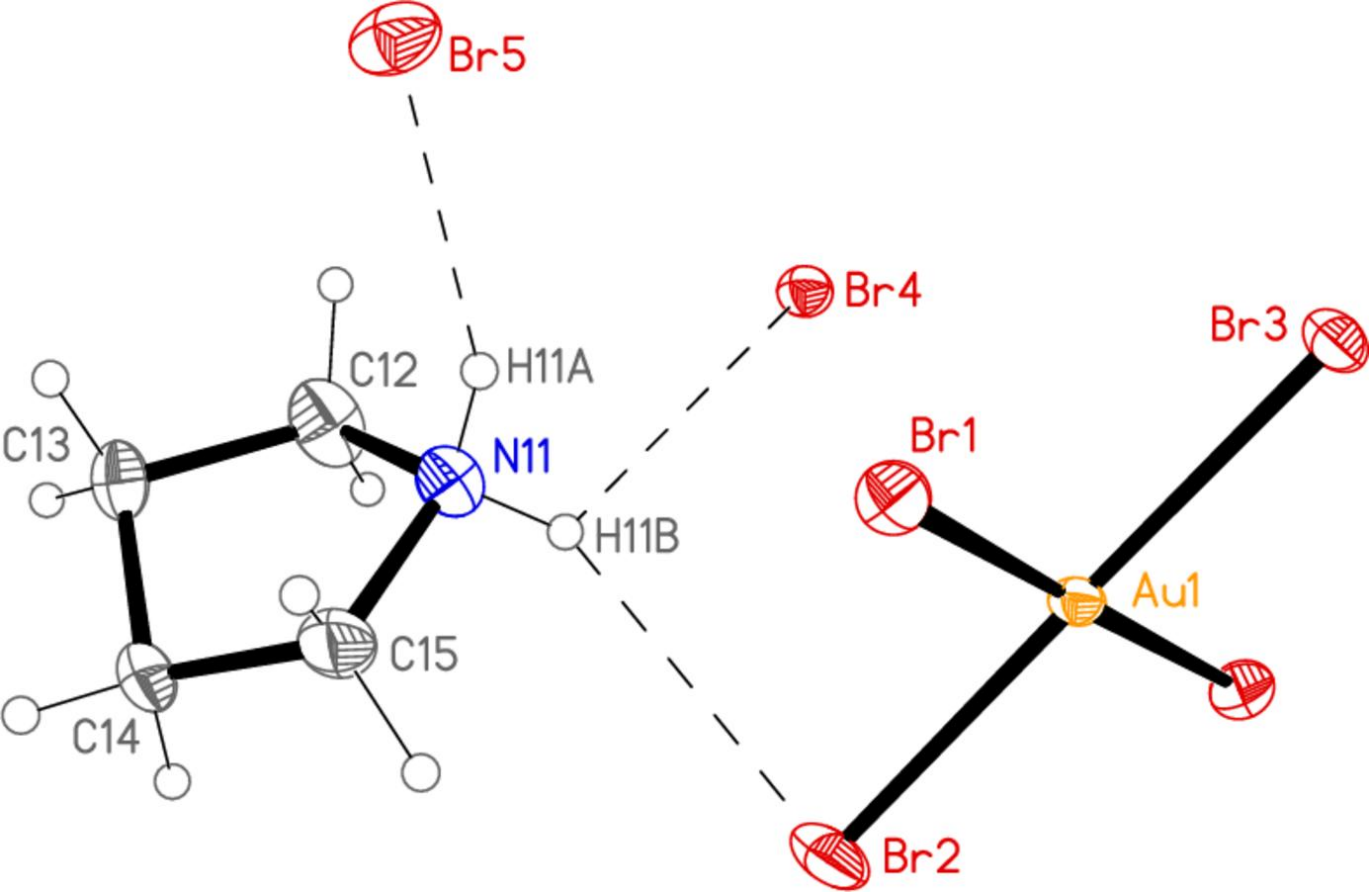
The asymmetric unit of compound **6**, extended by the symmetry-equivalent atom Br1′ (unlabelled), with ellipsoids at the 50% probability level. The dashed lines indicate hydrogen bonds.

The structure determination of compound **6** in space group *Ibam* proved to be relatively straightforward (but see section 6 below); the formula unit is shown in Fig. 6 ▸. The atoms Au1, Br2 and Br3 lie in the mirror plane *x*, *y*, 0; the bromide ions Br4 and Br5 lie on the special positions 0, 0.5, 0 and 0, 0.5, 0.25 respectively, with site symmetry 2/*m*. The pyrrolidinium ion lies on general positions. The [AuBr_4_]^−^ anion displays the expected square-planar geometry (Table 5 ▸). The NH_2_ group forms hydrogen bonds *via* H01 to the bromide ion Br5, and via H02 to form a three-centre system involving bromide Br4 and metal-bonded Br2 (Table 6 ▸); the H02⋯Br2 distance of 2.90 (6) Å is long, but three-centre distances and hydrogen bonds to metal-bonded halogens tend to be longer than conventional hydrogen bonds.

**Figure 7:**
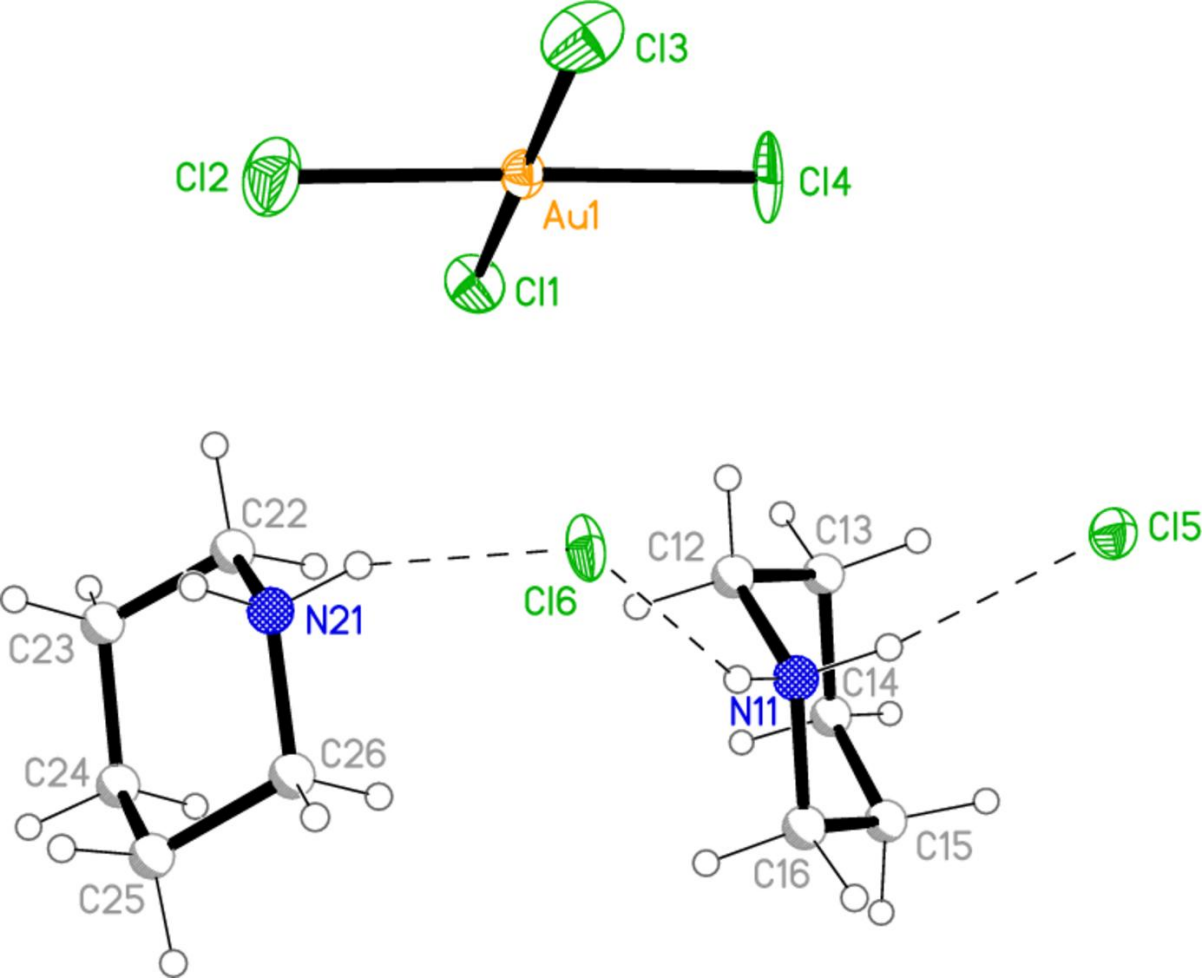
The asymmetric unit of compound **4** with ellipsoids at the 50% probability level. Because of the pseudosymmetry (see text), the carbon and hydrogen atoms had to be refined isotropically and are shown as circles of arbitrary radius. The dashed lines indicate hydrogen bonds.

The pseudosymmetric structure **4**, closely related to **6**, but crystallizing in space group *Iba*2, is shown in Fig. 7 ▸, with the dimensions of the anion in Table 7 ▸. Because of the reduced symmetry, all atoms occupy general positions apart from the chloride anions Cl5 and Cl6, which lie on the twofold axes 0.5, 0.5, *z*, and there are two independent cations. The significant shifts with respect to **6** mean that there is no longer a hydrogen bond from either NH_2_ group to a metal-bonded chlorine (Table 8 ▸).

**Figure 8:**
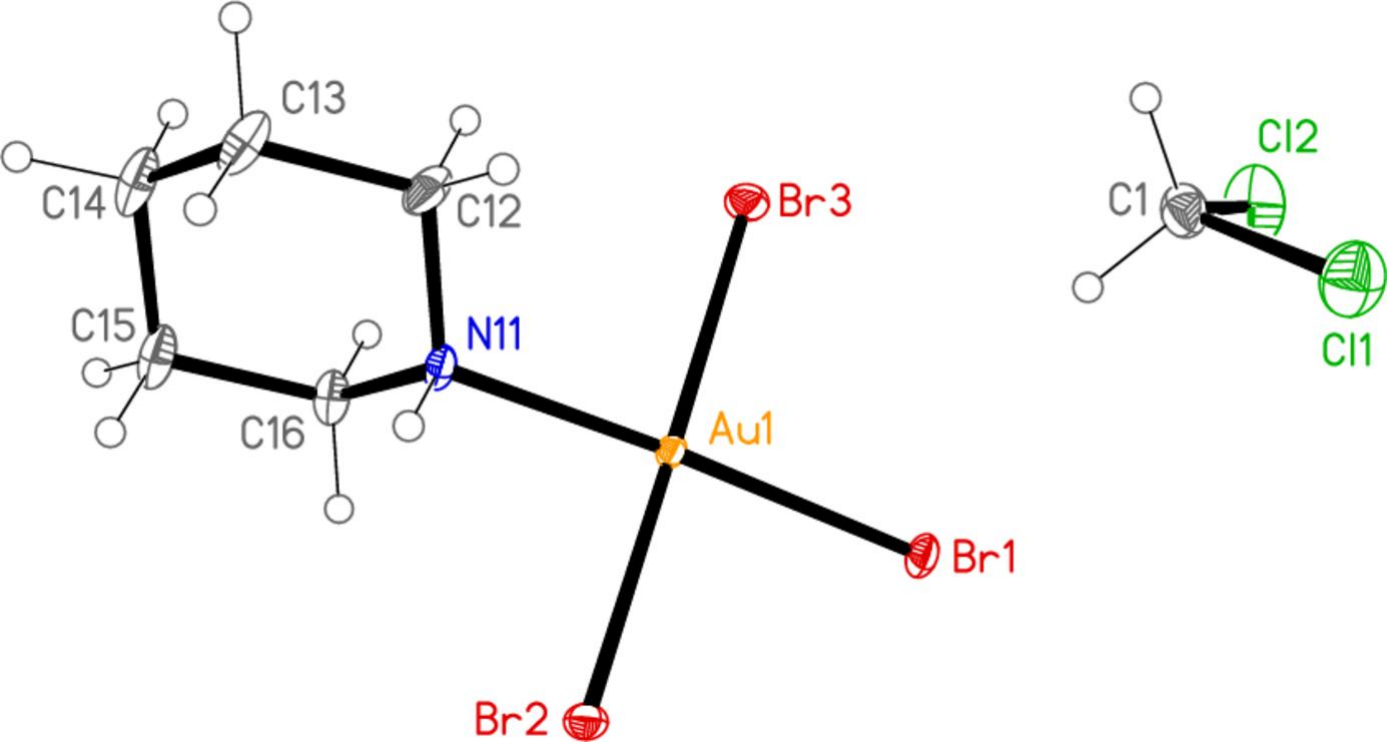
The asymmetric unit of compound **7** with ellipsoids at the 50% probability level.

The compound [AuBr_3_(pip)] crystallizes as its di­chloro­methane monosolvate **7** (Fig. 8 ▸); the solvent mol­ecule is well-ordered. The Au—Br1 bond *trans* to N11 is slightly shorter than the other Au—Br bonds (Table 9 ▸), but somewhat longer than in the [AuBr(pyr)] component of compound **2**. The r.m.s. deviation of Au1 and its bonded atoms from the plane that they define is 0.024 Å. In contrast to **3**, there is no intra­molecular hydrogen bond (Table 10 ▸) and the torsion angle H01—N11—Au1—Br2 is 31 (2)°. The anti­periplanar Au—N—C—C torsion angles again correspond to an equatorial position of the AuBr_3_ group at the piperidine ring.

## 3. Supra­molecular features

For the compounds consisting of more than one residue, supra­molecular features within the asymmetric units have already been discussed in the *Structural commentary*.

**Figure 9:**
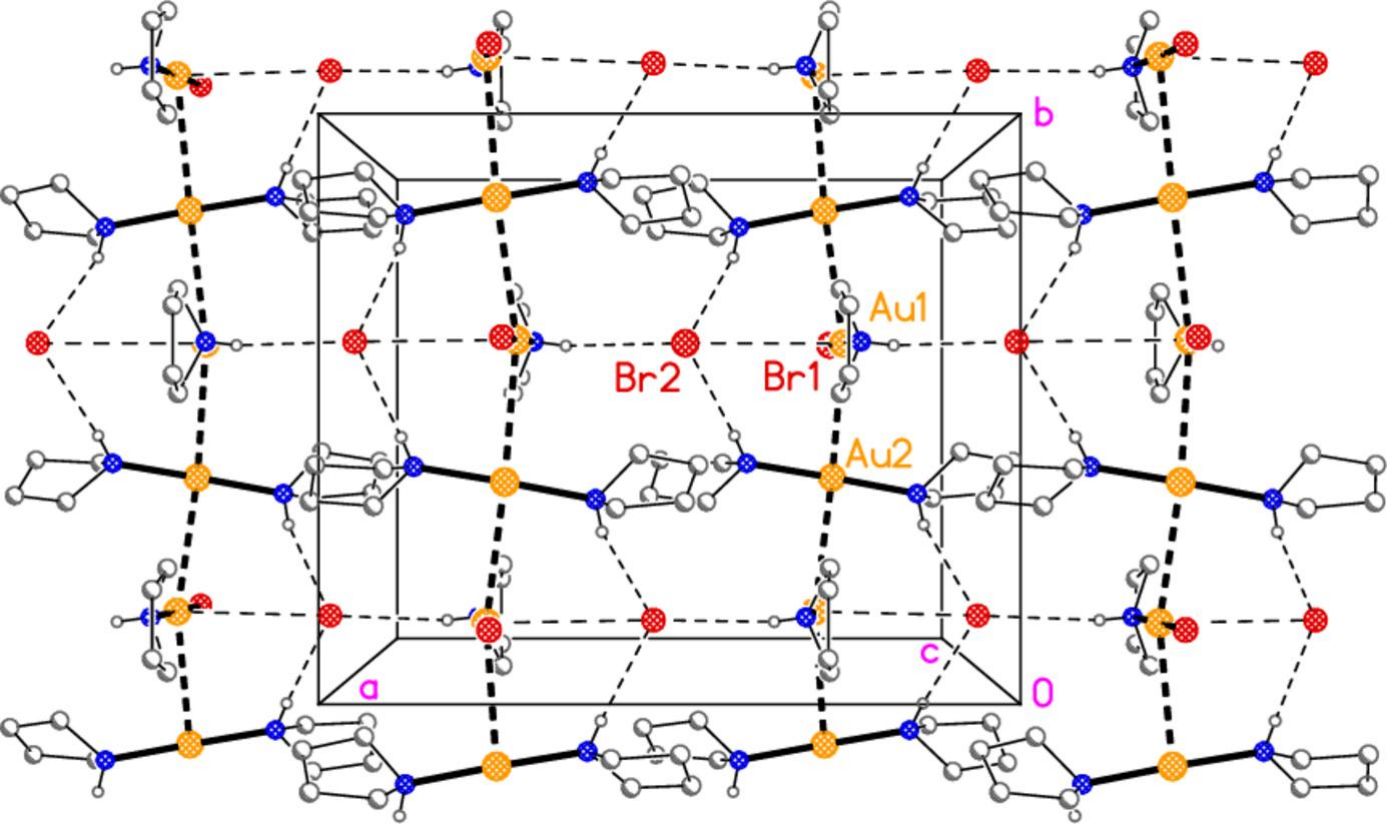
Packing diagram of compound **2** viewed parallel to the *c* axis in the region *z* ≃ 0.25. For all packing diagrams, hydrogen atoms bonded to carbon are omitted for clarity. Thin dashed lines indicate hydrogen bonds or Au1⋯Br2 inter­actions; thick dashed lines indicate aurophilic inter­actions. Note that the [AuBr(pyr)] mol­ecules (involving Au1) are viewed end-on. Labelled atoms belong to the asymmetric unit.

For compound **2**, there is a second aurophilic contact Au2⋯Au1 (at 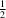 − *x*, − 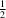 + *y*, *z*) 3.1549 (6) Å, so that the extended structure involves an infinite chain of alternating gold atoms Au1/Au2 (and thus of the corresponding residues), involving the *b* glide operator, with overall direction parallel to the *b* axis. The chain is almost linear, with Au⋯Au⋯Au angles of 163.07 (2) and 167.78 (2)°. The chains are cross-linked by N—H⋯Br2 hydrogen bonds (Table 2 ▸) and the borderline contacts Au1⋯Br2 [3.855 (1) Å], forming a layer structure (Fig. 9 ▸) parallel to the *ab* plane in the region *z* ≃ 0.25 (with a second such layer at *z* ≃ 0.75).

**Figure 10:**
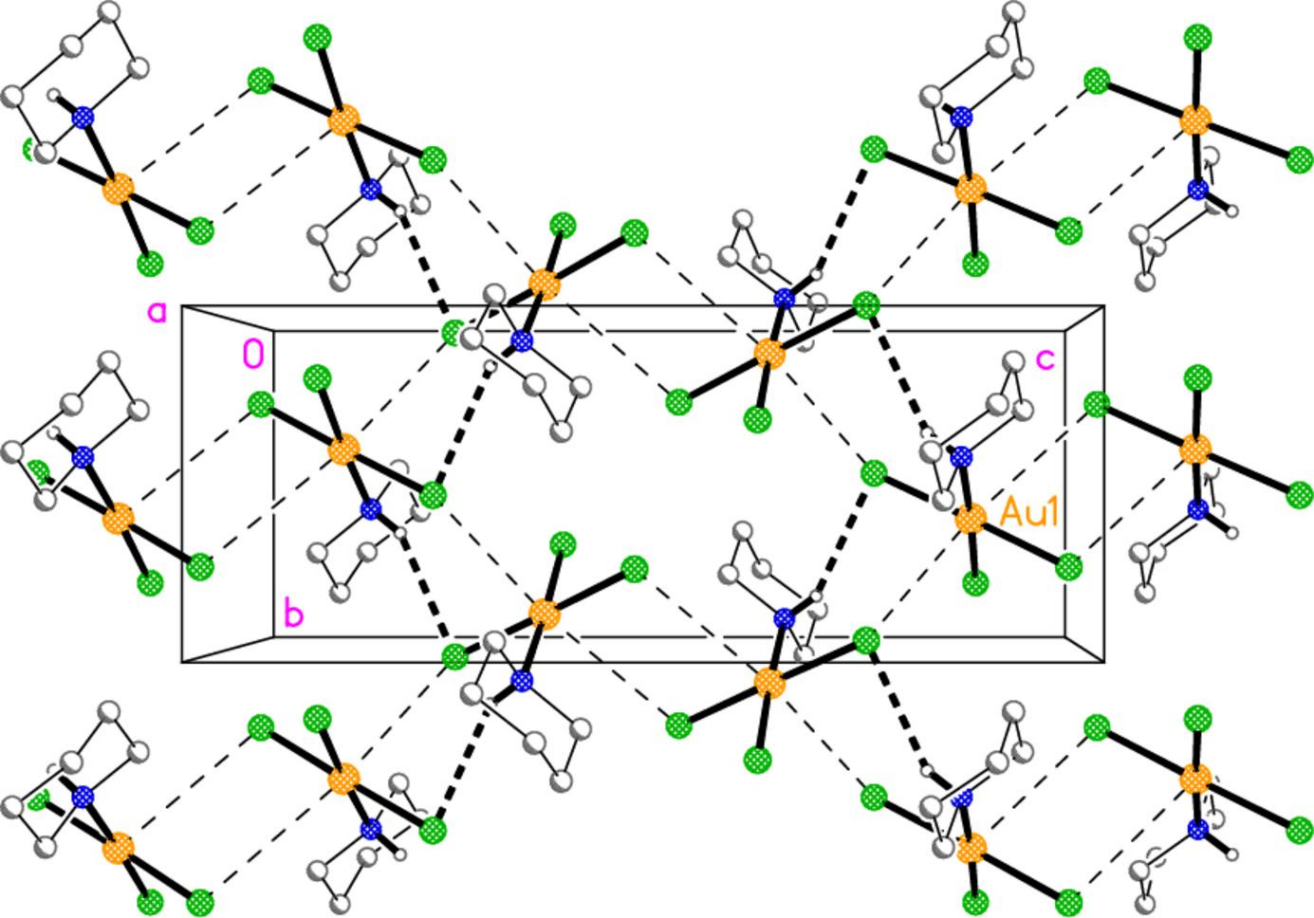
Packing diagram of compound **3** viewed perpendicular to the *bc* plane. Hydrogen atoms bonded to carbon are omitted for clarity. Thick dashed lines indicate hydrogen bonds; thin dashed lines indicate Au1⋯Cl3 inter­actions. The labelled atom belongs to the asymmetric unit.

For a compound such as **3**, the packing may involve a variety of secondary inter­actions, such as classical or ‘weak’ hydrogen bonds, Cl⋯Cl contacts (which represent a type of ‘halogen bond’; see *e.g.* Metrangolo *et al.*, 2008 ▸), or short Au⋯Cl contacts, sometimes leading to stacking of AuCl_3_ units, as observed for [AuCl_3_(tht)] (for which we determined the structures of four different forms; Upmann *et al.*, 2017 ▸) and for the primary amine complex [AuCl_3_(iso­propyl­amine)] (Döring & Jones, 2018*a* ▸). It is not always straightforward to establish objectively which contacts are more important, because packing patterns are determined by the energy of various inter­actions, whereas conventional structure deter­mination only gives distances between atoms. For **3** we subjectively assess the important effects to be classical hydrogen bonds and Au⋯Cl inter­actions; there are, however, several borderline C—H⋯Cl contacts that we do not consider (if only for the sake of simplicity). The hydrogen bond N11—H01⋯Cl3 (Table 4 ▸) is quite long, but acceptably linear, and the contact Au1⋯Cl3 (at 1 − *x*, 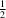 + *y*, 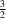 − *z*) at 3.3365 (6) Å is short. Both contacts involve *c* glide operators, and the overall effect is to form a layer structure parallel to the *bc* plane (Fig. 10 ▸). In each layer, the AuCl_3_ and NH_2_ units lie in the region *x* ≃ 0.5, and the rings project outwards from the layer, thus occupying the regions at *x* ≃ 0 and 1.

**Figure 11:**
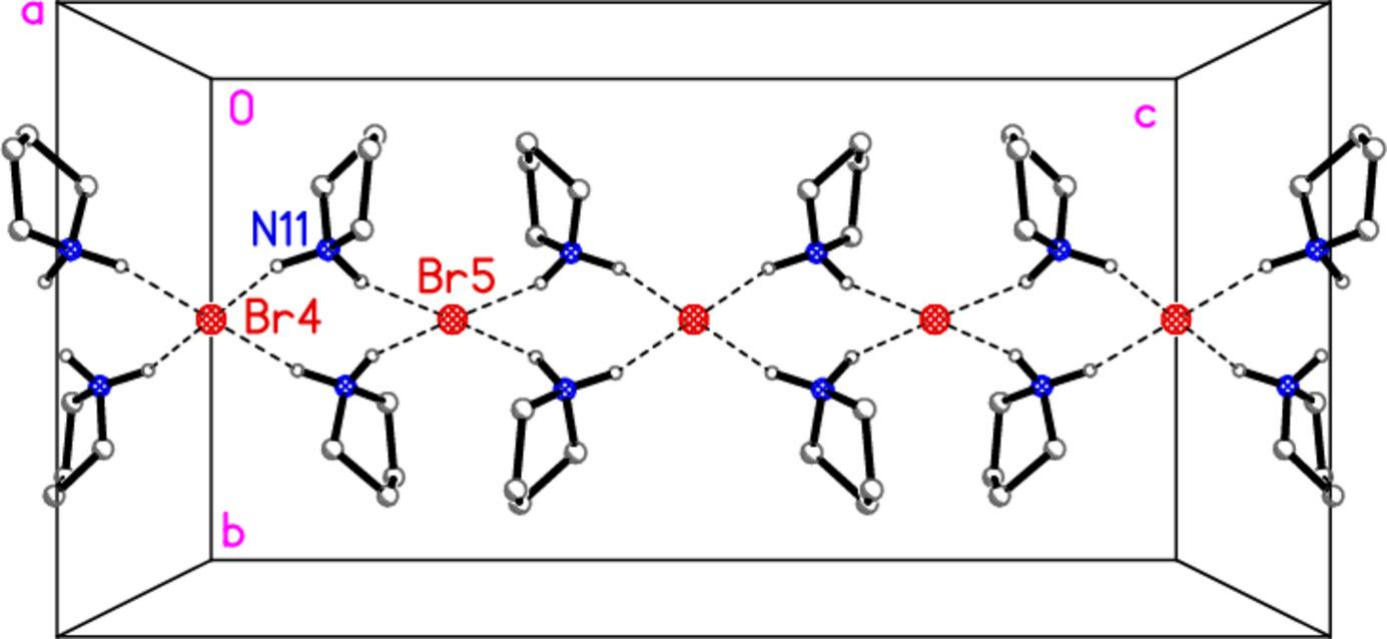
Packing of compound **6**, first substructure; the bromide and pyrrolidinium ions combine to form a chain parallel to the *c* axis. The view direction is parallel to the *a* axis. Dashed lines indicate hydrogen bonds. Labelled atoms belong to the asymmetric unit.

**Figure 12:**
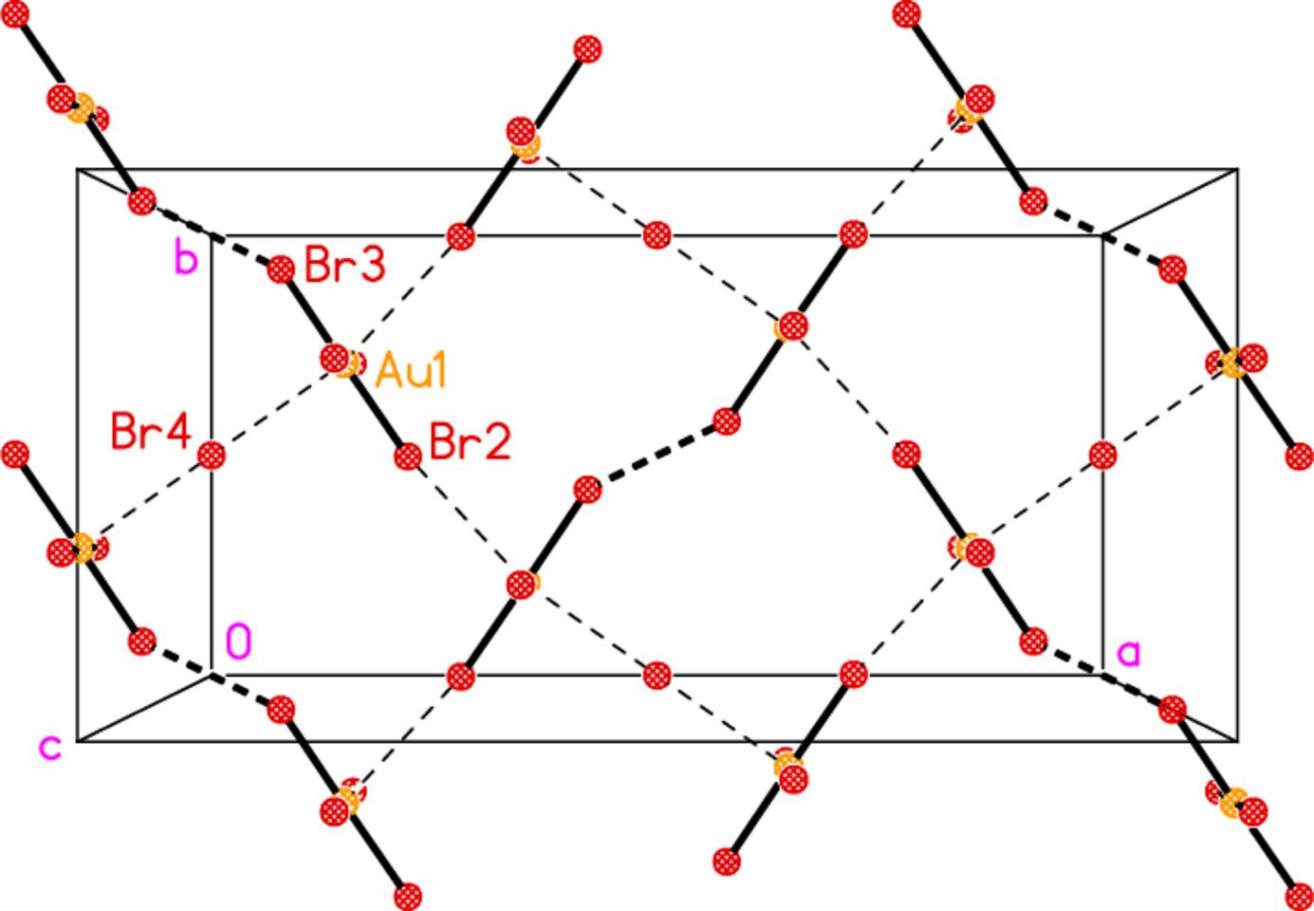
Packing of compound **6**, second substructure; the bromide ions Br4 and the tetra­bromido­aurate(III) ions (seen edge-on) combine to form a layer structure parallel to the *ab* plane. The view direction is parallel to the *c* axis, and the region shown is at *z* ≃ 0. Dashed lines indicate (thin) Au⋯Br or (thick) Br⋯Br inter­actions. Labelled atoms belong to the asymmetric unit.

The packing of compound **6** is complex, as might be expected in space group *Ibam*. However, it can be analysed in terms of two substructures. First, the bromide ions combine with the pyrrolidinium cations by classical hydrogen bonding (Table 6 ▸) to form a chain of residues parallel to the *c* axis (Fig. 11 ▸); the graph set of the hydrogen-bonded (NH_2_)_2_Br_2_ rings is 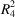 (8). Secondly, the [AuBr_4_]^−^ anions and the bromide Br4 combine to form a layer structure parallel to the *ab* plane (Fig. 12 ▸), with contacts Au1⋯Br4 = 3.4585 (3), Au1⋯Br2( 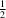 − *x*, 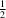 + *y*, *z*) = 3.6997 (8) and Br3⋯Br3(−*x*, 2 − *y*, −*z*) = 3.3201 (13) Å, with an associated Au—Br⋯Br angle of 149.92 (4)°. The two substructures are then linked by the hydrogen bonds involving the metal-bonded Br4 to complete the three-dimensional packing. Axial contacts to square-planar Au^III^ systems are well-known; the short Br⋯Br contact, however, might be considered unexpected between two anions (but see Section 4). We have presented several examples of such contacts between [Au*X*_4_]^−^ anions (*X* = Cl, Br) in a recent paper (Döring & Jones, 2016 ▸).

**Figure 13:**
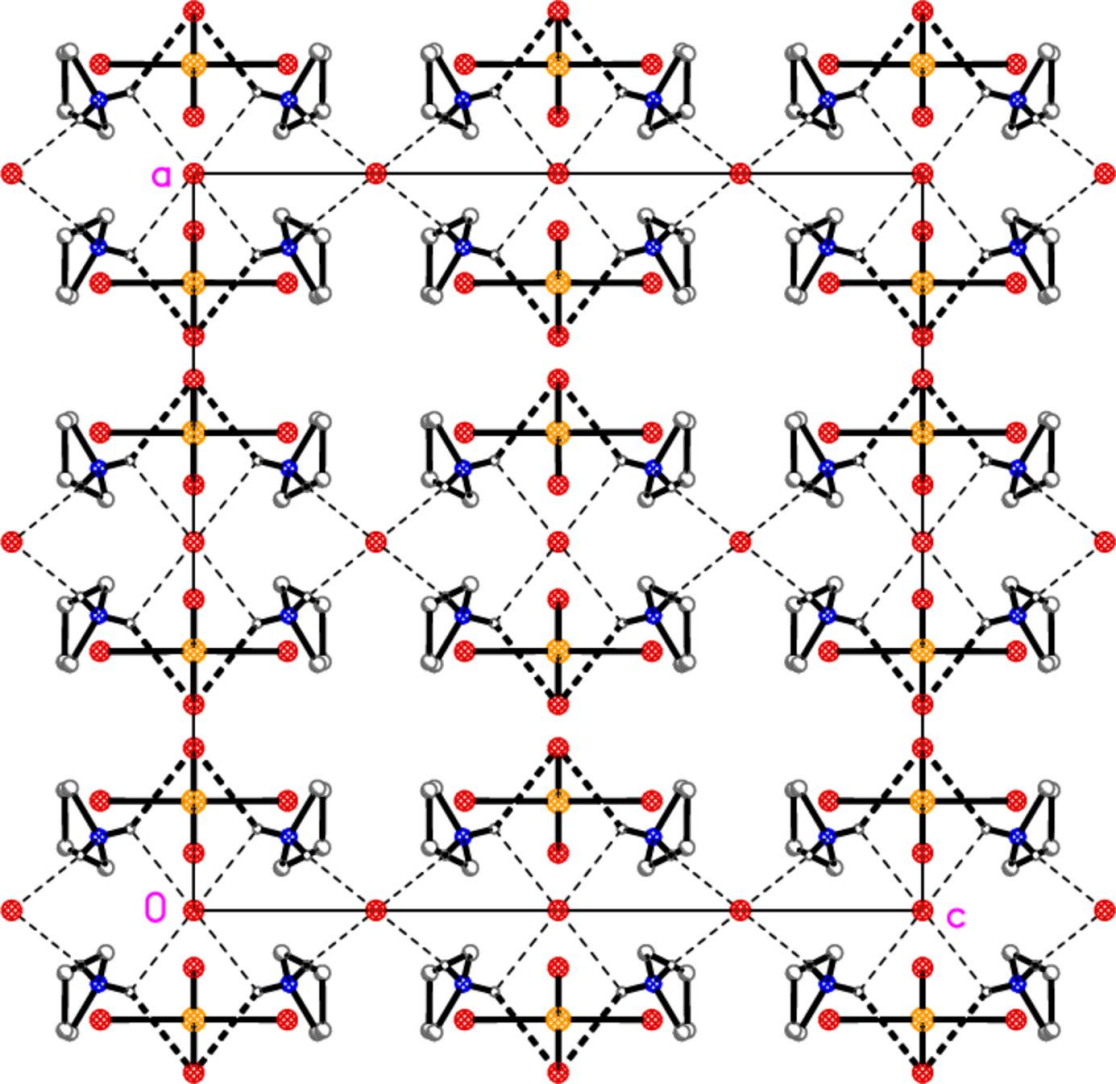
Projection of the structure of compound **6** parallel to the *b* axis. Thick dashed lines indicate the hydrogen bonds of the type N—H⋯Br—Au, thin dashed lines indicate other hydrogen bonds; other contacts are not explicitly included.

Similar substructures are present for compound **4** as for **6**, but there are significant differences. The chain of piperidinium and chloride ions in **4** is closely analogous to the pyrrolid­inium/bromide chain of **6**. The tetra­chlorido­aurate/chloride substructure is topologically closely similar to the tetra­bromido­aurate/bromide system of **6**, but the distances differ appreciably; thus the gold⋯chloride contacts Au1⋯Cl6(− 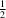 + *x*, 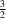 − *y*, *z*) = 3.8135 (4) Å and Au1⋯Cl1( 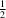 − *x*, − 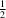 + *y*, *z*) = 3.995 (3) Å of **4** are, counter-intuitively, much longer than the corresponding Au⋯Br distances in **6** (and are probably too long to represent appreciable inter­actions), whereas the inter­anionic Cl3⋯Cl3(−*x*, 1 − *y*, *z*) contact of 3.085 (5) Å is much shorter than its Br⋯Br counterpart in **6**; the associated Au—Cl⋯Cl angle is wider than its counterpart in **6** at 168.1 (2)°. Qualitatively, the packing diagrams are the same as those presented in Figs. 11 ▸ and 12 ▸ for **6**, and so we do not present analogous diagrams for **4**. Another significant difference, as noted in Section 2, is that there are no N—H⋯Cl inter­actions involving a metal-bonded chloride; this is shown in projections of structures **6** and **4** parallel to their *b* axes (Figs. 13 ▸ and 14 ▸; these figures also show clearly the presence of mirror planes, perpendicular to the *c* axis, that relate pairs of cations in **6**, whereas this symmetry element is missing for the corres­ponding cation pairs in **4**). Instead, there are some short C—H⋯Cl contacts that may reasonably be considered as hydrogen bonds (Table 8 ▸). Compound **5** seems to be isotypic to **4**, but the pseudosymmetry proved too severe to refine the light atoms reliably. We observed similar effects in the structures of two closely related organic compounds, whereby a toluene­sulfonyl derivative crystallized in *P*2_1_/*c* with *Z*′ = 1 (Elgemeie *et al.*, 2013 ▸), whereas its benzene­sulfonyl analogue crystallized with a closely similar cell and structure in *Pc* with *Z*′ = 2 (Elgemeie *et al.*, 1998 ▸).

**Figure 15:**
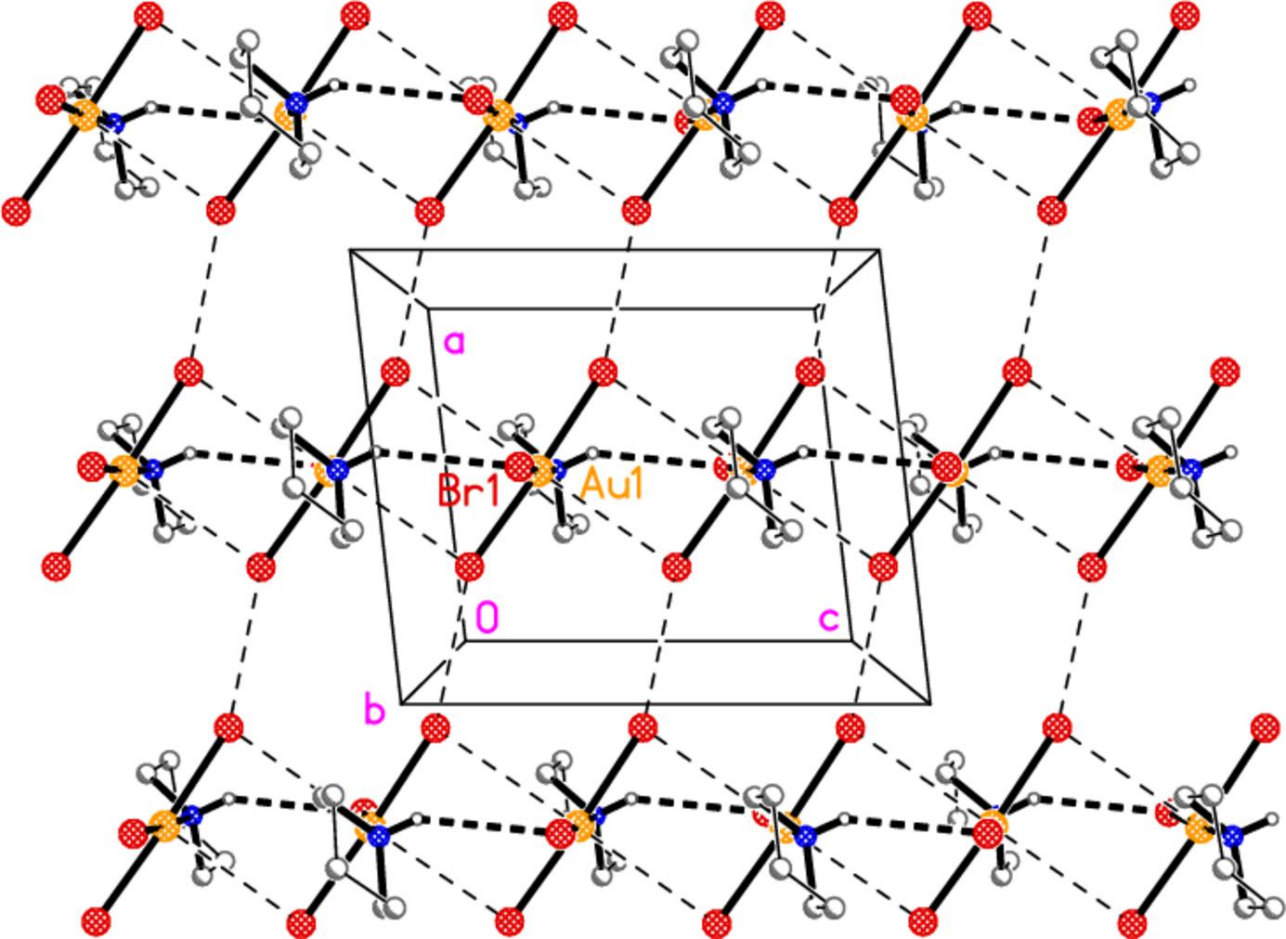
Packing of compound **7**. Solvent mol­ecules are omitted. The view direction is parallel to the *b* axis (so that the mol­ecules are seen approximately end-on), and the region shown is at *y* ≃ 0.25. Thick dashed lines indicate hydrogen bonds; thin dashed lines indicate Au⋯Br or Br⋯Br inter­actions. Labelled atoms belong to the asymmetric unit.

For compound **7**, classical hydrogen bonds (Table 10 ▸) connect the mol­ecules *via* a *c* glide operator to form chains parallel to the *c* axis (Fig. 15 ▸). These are reinforced by offset stacking of neighbouring AuBr_3_ units, with Au1⋯Br3(*x*, 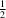 − *y*, 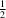 + *z*) = 3.4678 (4) Å and Au1⋯Br2(*x*, 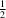 − *y*, − 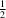 + *z*) = 3.5658 (4) Å. Finally, adjacent ribbons are connected by the contact Br2⋯Br3(1 + *x*, 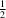 − *y*, 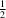 + *z*) = 3.3817 (4) Å to form a layer structure parallel to the *ac* plane at *y* ≃ 0.25. Another such layer lies at *y* ≃ 0.75. The di­chloro­methane mol­ecule is omitted from Fig. 15 ▸ for clarity; it forms a weak hydrogen bond to Br1 within the asymmetric unit and also displays a Cl1⋯Cl1 contact of 3.562 (2) Å to an adjacent solvent mol­ecule at −*x*, 1 − *y*, 1 − *z*.

## 4. Database survey

The searches employed the routine ConQuest (Bruno *et al.*, 2002 ▸), part of Version 2022.3.0 of the Cambridge Database (Groom *et al.*, 2016 ▸).

A search for all complexes of gold with unsubstituted piperidine or pyrrolidine ligands gave five hits for each; all of these were from our group. For the piperidine structures, one unexpected feature (that we failed to draw attention to at the time of the original publications) was a marked tendency for the gold moiety to lie axially with respect to the piperidine rings. Thus for [Au(pip)(SCN)] (refcode DIXBAQ; Strey *et al.*, 2018 ▸), [Au(pip)_2_][AgCl_2_] (DUHQUS/DUQHUS01; Ahrens *et al.*, 2000 ▸, corrected in Ahrens *et al.*, 2003 ▸), [Au(CN)(pip)] (FIMSOL; Döring & Jones, 2013 ▸) and [Au(pip)_2_]Cl (GOGFEN/GOGFEN01; Ahrens *et al.*, 1999 ▸), all the absolute C—C—N—Au torsion angles lie in the range 65–72°. A possible explanation might be that the low coordination number of gold alleviates the steric disadvantages somewhat. A more extensive search for piperidine complexes of any transition metal gave 193 hits, for most of which the metal residue lay equatorial to the piperidine ring. Almost all of the 35 exceptions belonged to the subset of 64 hits for coinage metals, with their generally low coordination numbers. For the pyrrolidine complexes, the situation was more clear-cut; 60 of the 63 hits had absolute *TM*—N—C—C torsion angles in the range 140–180°.

Searches for short inter­molecular Cl⋯Cl or Br⋯Br contacts in neutral complexes of the form [AuCl_3_ *L*] or [AuBr_3_ *L*] (as in compound **7**) were conducted. The Cl⋯Cl search gave 51 hits with distances up to 3.5 Å, twice the maximum (CCDC-defined) van der Waals radius, of which 24 were shorter than 3.4 Å. The shortest were 3.086 and 3.191 Å between *cis* (to *L*) chlorines in two carbene complexes (HOLGUM and HOKJIC; Teci *et al.*, 2017 ▸ and Tomás-Mendivil *et al.*, 2013 ▸). The Br⋯Br search gave 28 hits up to 3.7 Å; 11 were shorter than 3.6 Å. The shortest was 3.260 Å between *cis* bromines in a phosphine sulfide complex (BOKQUQ; Upmann *et al.*, 2019 ▸).

## 5. Synthesis and crystallization

Syntheses were performed under an atmosphere of dry nitro­gen; the small-scale crystallization experiments were performed in laboratory air.

*Bromido­(piperidine)­gold(I) bis­(piperidine)­gold(I) bromide (**1**)*: 90 mg (0.247 mmol) [AuBr(tht)] were dissolved in 2 mL piperidine. The solution was divided into five small test tubes and overlayered with five different precipitants. The test-tubes were stoppered and stored in a refrigerator at 278 K for 1 day. Crystals in the form of colourless laths were obtained in small qu­anti­ties using petroleum ether as precipitant, despite considerable decomposition that led to a gold mirror.

*Bromido­(pyrrolidine)gold(I) bis­(pyrrolidine)gold(I) bro­mide (**2**)*: 45 mg (0.123 mmol) [AuBr(tht)] were dissolved in 2 mL pyrrolidine. Crystals were obtained as for **1**, but with diisopropyl ether as precipitant.

*Tri­chlorido­(piperidine)­gold(III) (**3**)* and *bis­(piperidinium) chloride tetra­chlorido­aurate(III) (**4**)*: 120 mg (0.374 mmol) [AuCl(tht)] were dissolved in a mixture of 4 mL of piperidine and 4 mL of di­chloro­methane. The solution was overlayered with *n*-pentane in a 100 mL round-bottomed flask and transferred to the refrigerator overnight. The supernatant was pipetted off and the solid residue (presumed to be [Au(pip)_2_]Cl) dried *in vacuo* (148.7 mg, 0.369 mmol). The solid was divided into two parts; each was dissolved in 2 mL of di­chloro­methane, and 50.7 mg (0.184 mmol) of PhICl_2_ in 2 mL of di­chloro­methane was added to each, causing the solutions to turn first red and then orange. After 16 days at 278 K, crystals of **3** (yellow blocks and laths, 91% yield) were obtained using *n*-heptane as precipitant, and of **4** (a few yellow laths) using petroleum ether.

*Bis(piperidinium) bromide tetra­bromido­aurate(III) (**5**)*: 49.6 mg (0.136 mmol) [AuBr(tht)] were dissolved in 2 mL piperidine and overlayered with *n*-pentane in a test-tube, which was stoppered and transferred to the refrigerator overnight. The supernatant was pipetted off and the solid residue dried *in vacuo* to give 541 mg (0.067 mmol, 98%) of compound **1**. This was dissolved in 2 mL of di­chloro­methane, and 2 drops of elemental bromine were added. The solution was overlayered with diisopropyl ether and stored at 278 K for 4 days, leading to crystals in the form of red plates (78% yield). Elemental analysis [%]: calc.: C 15.62, H 3.15, N 3.64; found: C 15.68, H 3.32, N 3.86.

*Bis(pyrrolidinium) bromide tetra­bromido­aurate(III) (**6**)*: 135.7 mg (0.372 mmol) of [AuBr(tht)] were dissolved in 2 mL of pyrrolidine. Diisopropyl ether was added until a permanent turbidity was observed, and the mixture was transferred to the refrigerator overnight. The supernatant was pipetted off and the solid dark-grey residue was taken up in 4 mL of di­chloro­methane. After filtration, two drops of elemental bromine were added, leading to a dark-red solution with a dark-red solid residue (not identified). After this had settled, the clear solution was pipetted off into five test-tubes and overlayered as above for **1**. After 1 day at 278 K, crystals in the form of dark-red tablets and laths (yield not determined) were obtained with diisopropyl ether as precipitant.

*Tri­bromido­(piperidine)­gold(III) di­chloro­methane solvate (**7**)*: 127 mg (0.157 mmol) of compound **1** were dissolved in 4 mL of di­chloro­methane, and two drops of elemental bromine were added. 2 mL of the solution were subjected to five different precipitants as described above for **1**; after 10 days at 278 K, crystals in the form of orange–red needles (yield not determined) were obtained using *n*-heptane as precipitant. Elemental analysis [%]: calculated (including the solvent content): C 11.88, H 2.16, N 2.31; found: C 11.83, H 2.19, N 3.15.

## 6. Refinement

Crystal data, data collection and structure refinement details are summarized in Table 11 ▸. Structures were refined anisotropically on *F* ^2^. Hydrogen atoms of the NH groups were refined freely. Methyl­ene hydrogens were included at calculated positions and refined using a riding model with C—H = 0.99 Å and H—C—H = 109.5°. Isotropic *U*(H) values were fixed at 1.2 × *U*_eq_ of the parent carbon atom (or nitro­gen, see below).

*Special details and exceptions*: The structure of compound **2** was difficult to refine satisfactorily; the data are weak and the absorption coefficient is high. Hydrogen atoms of the NH groups were located in difference syntheses and refined freely, but with N—H distances restrained to be approximately equal (command ‘SADI’); the positions of freely refined hydrogen atoms in heavy-atom structures should of course be inter­preted with caution, but seem to be acceptable for **2** and for the other structures presented here. The ring at N31 is disordered, with atoms C33 and C34 occupying alternative sites with occupation factors 0.64 (2) and 0.36 (2); atoms of the minor site were refined isotropically. Appropriate restraints were employed to improve refinement stability, but the dimensions of disordered groups should always be inter­preted with caution. The residual electron density near the gold atom was high, which is probably attributable to residual absorption errors; for poor data, errors are likely to be reflected in this way. Nevertheless we believe that the refinement provides at least a qualitatively reliable picture of the structure. Data for compound **1** were also collected, showing that it is effectively isotypic to **2**, but the refinement was highly unsatisfactory, with two very large difference peaks that did not lie close to the gold atom. Despite considerable efforts, we were unable either to explain these peaks (*e.g*. by detecting additional weak reflections corresponding to a larger cell or indicating twinning effects) or to collect better data from other crystals of **1** and **2**.

For compounds **3** and **6**, extinction corrections were performed using the command ‘EXTI’; the extinction parameters (as defined by *SHELXL*; Sheldrick, 2015 ▸) refined to 0.00109 (7) and 0.00041 (2) respectively.

The structure of compound **4**, which is pseudosymmetric, was refined as a two-component inversion twin; the relative volume of the smaller component refined to 0.45 (3). Originally the structure was refined in space group *Ibam*, whereby the gold atom and four of the five chlorine atoms lay in mirror planes; the piperidinium cations were disordered. However, it can be refined with ordered cations in *Iba*2, so we prefer this model. Because of the well-known difficulties of refining an almost centrosymmetric structure in a non-centrosymmetric space group, the light atoms (carbon and nitro­gen) had to remain isotropic, and many restraints were necessary to improve refinement stability. The dimensions of the cations should therefore be inter­preted with caution. There is also the danger that the refinement results may represent a false minimum (although these often involve chemically implausible structures). The hydrogen atoms, in particular those of the NH_2_ groups, could not be located in difference syntheses and were therefore included using a riding model starting from calculated positions (with N—H 0.91 Å). The closely related structure of compound **6**, however, was successfully refined in *Ibam* without disorder. The hydrogen atoms of the NH_2_ group were refined freely, but with N—H distances restrained to be approximately equal (command ‘SADI’). We also recorded a dataset for **5**, which appears to be isotypic to **4**, but for which the pseudosymmetry proved too severe to allow satisfactory refinement.

We note also that, for rings of the form [(CH_2_)_n_NH_2_]^+^, it may be difficult to distinguish between the carbon and nitro­gen atoms in the presence of heavy atoms (especially for pseudosymmetric structures such as **4**). Our assignments of these atoms were based on *U* values (although these are somewhat irregular, *e.g.* the low value of 0.014 Å^2^ for C14 of **4**) and, more importantly, on the hydrogen-bonding patterns of the corresponding hydrogen atoms; thus only the hydrogen atoms of the chosen nitro­gen sites are involved in the short hydrogen-halide contacts of the cation/halide chains of **4** and **6**. However, the H⋯Br distances for hydrogen bonds N—H⋯Br and C—H⋯Br are unlikely to differ greatly, so some degree of C/N disorder for **6** cannot be ruled out.

For compound **7**, *checkCIF* suggested a smaller cell, generated by halving the *b* axis. However, careful inspection of the data shows that the reported cell is correct. Reflections with *k* odd are weaker, but definitely present. We note that the gold atom and two of the three bromine atoms have *y* coordinates of approximately 0.25; this is probably the factor responsible for the systematically weak reflections.

## Supplementary Material

Crystal structure: contains datablock(s) 2, 3, 4, 6, 7, global. DOI: 10.1107/S205698902300854X/yz2040sup1.cif

Structure factors: contains datablock(s) 2. DOI: 10.1107/S205698902300854X/yz20402sup2.hkl

Structure factors: contains datablock(s) 3. DOI: 10.1107/S205698902300854X/yz20403sup3.hkl

Structure factors: contains datablock(s) 4. DOI: 10.1107/S205698902300854X/yz20404sup4.hkl

Structure factors: contains datablock(s) 6. DOI: 10.1107/S205698902300854X/yz20406sup5.hkl

Structure factors: contains datablock(s) 7. DOI: 10.1107/S205698902300854X/yz20407sup6.hkl

CCDC references: 2113940, 2113939, 2113938, 2113937, 2297985

Additional supporting information:  crystallographic information; 3D view; checkCIF report

## Figures and Tables

**Figure 14:**
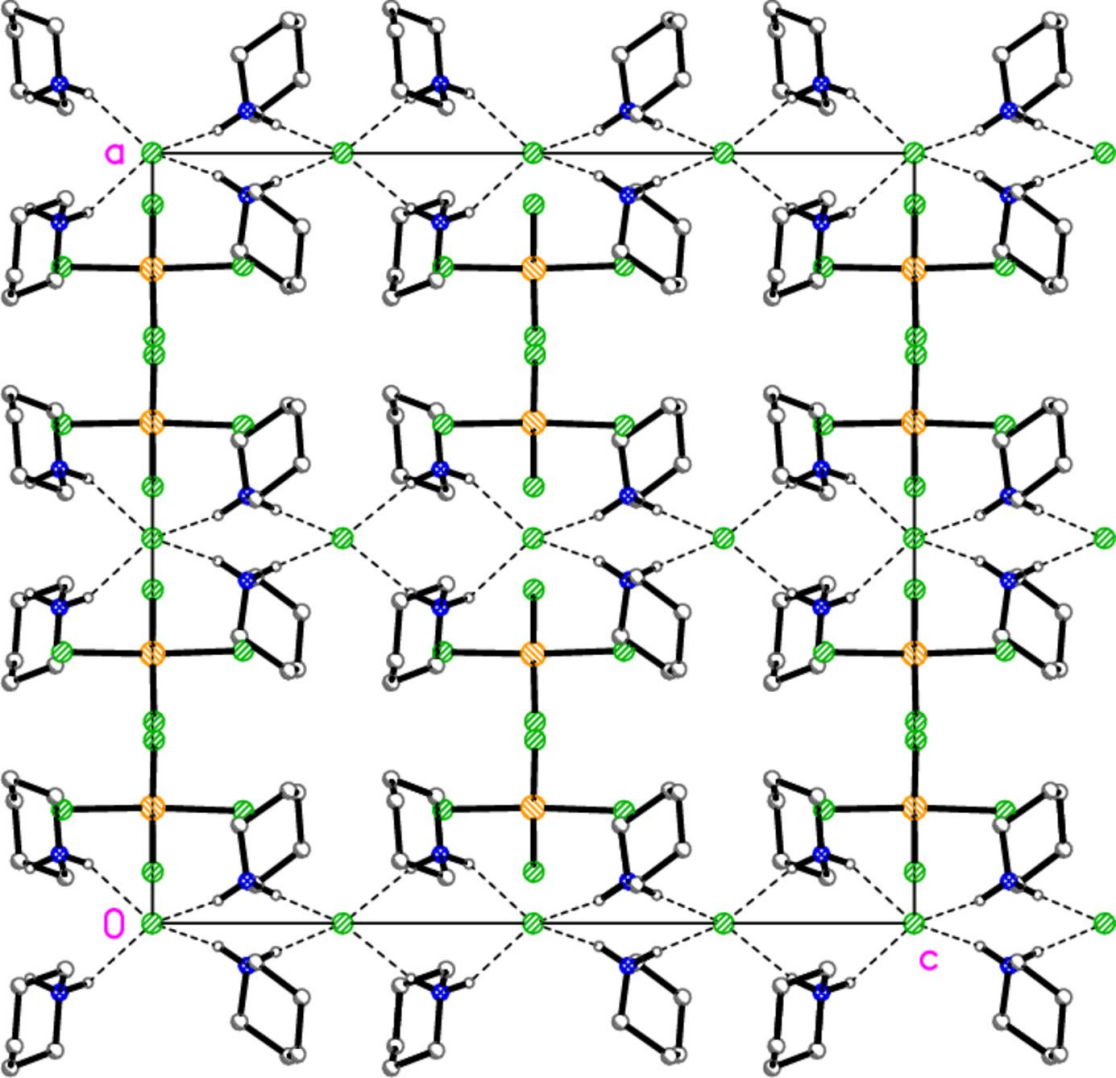
Projection of the structure of compound **4** parallel to the *b* axis. Dashed lines indicate hydrogen bonds; other contacts are not explicitly included. Note the absence of hydrogen bonds of the type N—H⋯Cl—Au (*cf.* Fig. 13 ▸).

**Table 1:** Selected geometric parameters (Å, °) for **2** [Chem scheme1]

Au1—N11	2.065 (9)	Au1—Au2^i^	3.1551 (6)
Au1—Br1	2.3837 (12)	Au2—N21	2.021 (11)
Au1—Au2	3.1476 (6)	Au2—N31	2.036 (12)
			
N11—Au1—Br1	176.3 (3)	N21—Au2—N31	175.4 (4)
Au2—Au1—Au2^i^	163.074 (19)	Au1—Au2—Au1^ii^	167.78 (2)
			
Br1—Au1—Au2—N21	−83.2 (3)	Br1—Au1—Au2—N31	92.4 (3)

Symmetry codes: (i) 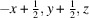 ; (ii) 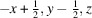 .

**Table 2:** Hydrogen-bond geometry (Å, °) for **2** [Chem scheme1]

*D*—H⋯*A*	*D*—H	H⋯*A*	*D*⋯*A*	*D*—H⋯*A*
N11—H01⋯Br2^iii^	0.81 (7)	2.70 (7)	3.498 (9)	169 (9)
N21—H02⋯Br2	0.81 (7)	2.69 (8)	3.477 (10)	162 (11)
N31—H03⋯Br2^ii^	0.81 (7)	2.68 (10)	3.416 (12)	151 (14)

Symmetry codes: (ii) 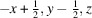 ; (iii) 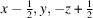 .

**Table 3:** Selected geometric parameters (Å, °) for **3** [Chem scheme1]

Au1—N11	2.0718 (19)	Au1—Cl3	2.2817 (6)
Au1—Cl2	2.2757 (6)	Au1—Cl1	2.2868 (6)
			
N11—Au1—Cl2	91.19 (6)	Cl2—Au1—Cl1	90.77 (2)
N11—Au1—Cl3	87.73 (6)	Cl3—Au1—Cl1	90.25 (2)
Cl2—Au1—Cl3	177.91 (2)	C16—N11—C12	112.49 (19)
N11—Au1—Cl1	177.36 (6)		
			
Au1—N11—C12—C13	179.30 (16)	Au1—N11—C16—C15	−176.54 (17)

**Table 4:** Hydrogen-bond geometry (Å, °) for **3** [Chem scheme1]

*D*—H⋯*A*	*D*—H	H⋯*A*	*D*⋯*A*	*D*—H⋯*A*
N11—H01⋯Cl3	0.86 (3)	2.57 (3)	3.021 (2)	114 (2)
N11—H01⋯Cl3^i^	0.86 (3)	2.79 (3)	3.558 (2)	150 (3)

Symmetry code: (i) 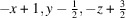 .

**Table 5:** Selected geometric parameters (Å, °) for **6** [Chem scheme1]

Au1—Br3	2.4102 (7)	Au1—Br2	2.4393 (8)
Au1—Br1	2.4303 (5)		
			
Br3—Au1—Br1	89.399 (13)	Br3—Au1—Br2	179.29 (3)
Br1—Au1—Br1^i^	178.15 (3)	Br1—Au1—Br2	90.592 (13)

Symmetry code: (i) 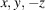 .

**Table 6:** Hydrogen-bond geometry (Å, °) for **6** [Chem scheme1]

*D*—H⋯*A*	*D*—H	H⋯*A*	*D*⋯*A*	*D*—H⋯*A*
N11—H01⋯Br5	1.03 (4)	2.26 (5)	3.253 (5)	161 (5)
N11—H02⋯Br2	1.02 (4)	2.90 (6)	3.624 (5)	128 (5)
N11—H02⋯Br4	1.02 (4)	2.65 (6)	3.398 (5)	130 (5)
C12—H12*A*⋯Br3^ii^	0.99	2.87	3.691 (6)	141

Symmetry code: (ii) 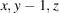 .

**Table 7:** Selected geometric parameters (Å, °) for **4** [Chem scheme1]

Au1—Cl3	2.267 (3)	Au1—Cl4	2.284 (11)
Au1—Cl2	2.278 (13)	Au1—Cl1	2.287 (3)
			
Cl3—Au1—Cl2	89.8 (4)	Cl3—Au1—Cl1	177.0 (3)
Cl3—Au1—Cl4	88.2 (4)	Cl2—Au1—Cl1	91.6 (3)
Cl2—Au1—Cl4	176.70 (12)	Cl4—Au1—Cl1	90.6 (4)

**Table 8:** Hydrogen-bond geometry (Å, °) for **4** [Chem scheme1]

*D*—H⋯*A*	*D*—H	H⋯*A*	*D*⋯*A*	*D*—H⋯*A*
N11—H11*A*⋯Cl6	0.91	2.32	3.150 (16)	151
N11—H11*B*⋯Cl5	0.91	2.26	3.161 (16)	172
C16—H16*A*⋯Cl3^i^	0.99	2.68	3.601 (16)	155
N21—H22*B*⋯Cl6	0.91	2.31	3.150 (15)	153
N21—H22*A*⋯Cl5^ii^	0.91	2.35	3.246 (17)	166
C23—H24*A*⋯Cl2	0.99	2.60	3.47 (2)	148

Symmetry codes: (i) 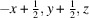 ; (ii) 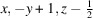 .

**Table 9:** Selected geometric parameters (Å, °) for **7** [Chem scheme1]

Au1—N11	2.091 (3)	Au1—Br3	2.4270 (3)
Au1—Br1	2.4187 (4)	N11—C16	1.495 (4)
Au1—Br2	2.4260 (3)	N11—C12	1.497 (4)
			
N11—Au1—Br1	177.24 (8)	Br1—Au1—Br3	88.920 (12)
N11—Au1—Br2	86.35 (8)	Br2—Au1—Br3	177.454 (13)
Br1—Au1—Br2	90.913 (12)	C16—N11—C12	111.7 (3)
N11—Au1—Br3	93.80 (8)		
			
Au1—N11—C12—C13	−176.2 (2)	Au1—N11—C16—C15	173.2 (2)

**Table 10:** Hydrogen-bond geometry (Å, °) for **7** [Chem scheme1]

*D*—H⋯*A*	*D*—H	H⋯*A*	*D*⋯*A*	*D*—H⋯*A*
N11—H01⋯Br1^i^	0.85 (4)	2.87 (4)	3.627 (3)	148 (3)
C14—H14*A*⋯Br1^ii^	0.99	2.82	3.788 (4)	167
C1—H1*A*⋯Br1	0.99	2.87	3.765 (4)	150

Symmetry codes: (i) 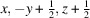 ; (ii) 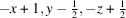 .

**Table 11:** Experimental details

	**2**	**3**	**4**	**6**	**7**
Crystal data					
Chemical formula	[AuBr(C_4_H_9_N)]·[Au(C_4_H_9_N)_2_]Br	[AuCl_3_(C_5_H_11_N)]	(C_5_H_12_N)_2_[AuCl_4_]Cl	(C_4_H_10_N)_2_[AuBr_4_]Br	[AuBr_3_(C_5_H_11_N)]·CH_2_Cl_2_
*M*_r_	767.12	388.46	546.53	740.78	606.77
Crystal system, space group	Orthorhombic, *P* *b* *c* *a*	Monoclinic, *P*2_1_/*c*	Orthorhombic, *I* *b* *a*2	Orthorhombic, *I* *b* *a* *m*	Monoclinic, *P*2_1_/*c*
Temperature (K)	100	100	100	100	100
*a*, *b*, *c* (Å)	14.8040 (11), 12.4631 (6), 19.4486 (8)	8.47646 (16), 6.57436 (11), 16.9961 (3)	19.4014 (15), 9.7612 (6), 19.1922 (11)	19.1275 (7), 9.4396 (13), 18.9259 (17)	7.3473 (3), 22.0860 (8), 8.5066 (3)
α, β, γ (°)	90, 90, 90	90, 93.5133 (16), 90	90, 90, 90	90, 90, 90	90, 96.423 (3), 90
*V* (Å^3^)	3588.3 (3)	945.36 (3)	3634.6 (4)	3417.2 (6)	1371.71 (9)
*Z*	8	4	8	8	4
Radiation type	Mo *K*α	Mo *K*α	Mo *K*α	Mo *K*α	Mo *K*α
μ (mm^−1^)	20.78	16.34	8.82	20.28	19.82
Crystal size (mm)	0.08 × 0.08 × 0.01	0.17 × 0.17 × 0.15	0.10 × 0.05 × 0.03	0.15 × 0.12 × 0.03	0.15 × 0.12 × 0.10

Data collection					
Diffractometer	Oxford Diffraction Xcalibur, Eos	Oxford Diffraction Xcalibur, Eos	Oxford Diffraction Xcalibur, Eos	Oxford Diffraction Xcalibur, Eos	Oxford Diffraction Xcalibur, Eos
Absorption correction	Multi-scan (*CrysAlis PRO*; Agilent, 2014 ▸)	Multi-scan (*CrysAlis PRO*; Agilent, 2014 ▸)	Multi-scan (*CrysAlis PRO*; Agilent, 2014 ▸)	Multi-scan (*CrysAlis PRO*; Agilent, 2014 ▸)	Multi-scan (*CrysAlis PRO*; Agilent, 2014 ▸)
*T*_min_, *T*_max_	0.287, 0.819	0.689, 1.000	0.718, 1.000	0.177, 1.000	0.574, 1.000
No. of measured, independent and observed [*I* > 2σ(*I*)] reflections	129826, 4461, 3055	24604, 2853, 2690	19554, 4242, 2632	35669, 2689, 2205	36043, 4139, 3658
*R*_int_	0.143	0.037	0.075	0.085	0.043
(sin θ/λ)_max_ (Å^−1^)	0.667	0.727	0.666	0.724	0.723

Refinement					
*R*[*F* ^2^ > 2σ(*F* ^2^)], *wR*(*F* ^2^), *S*	0.051, 0.130, 1.03	0.017, 0.030, 1.13	0.041, 0.067, 1.02	0.034, 0.065, 1.08	0.024, 0.039, 1.15
No. of reflections	4461	2853	4242	2689	4139
No. of parameters	193	96	105	88	123
No. of restraints	90	0	59	1	0
H-atom treatment	H atoms treated by a mixture of independent and constrained refinement	H atoms treated by a mixture of independent and constrained refinement	H-atom parameters constrained	H atoms treated by a mixture of independent and constrained refinement	H atoms treated by a mixture of independent and constrained refinement
Δρ_max_, Δρ_min_ (e Å^−3^)	7.82, −2.45	0.92, −0.80	0.84, −0.86	2.21, −1.24	0.84, −0.83
Absolute structure	–	–	Refined as an inversion twin	–	–
Absolute structure parameter	–	–	0.45 (3)	–	–

Computer programs: *CrysAlis PRO* (Agilent, 2014 ▸), *SHELXS97* (Sheldrick, 2008 ▸), *SHELXL2018/3* (Sheldrick, 2015 ▸) and *XP* (Siemens, 1994 ▸).

## supplementary crystallographic information

### Bromido(pyrrolidine-κN)gold(I) bis(pyrrolidine-κN)gold(I) bromide (2) Crystal data

**Table:**

[AuBr(C_4_H_9_N)]·[Au(C_4_H_9_N)_2_]Br	*D*_x_ = 2.840 Mg m^−^^3^
*M**_r_* = 767.12	Mo *K*α radiation, λ = 0.71073 Å
Orthorhombic, *P**b**c**a*	Cell parameters from 7080 reflections
*a* = 14.8040 (11) Å	θ = 2.5–29.3°
*b* = 12.4631 (6) Å	µ = 20.78 mm^−^^1^
*c* = 19.4486 (8) Å	*T* = 100 K
*V* = 3588.3 (3) Å^3^	Plate, colourless
*Z* = 8	0.08 × 0.08 × 0.01 mm
*F*(000) = 2784	

### Bromido(pyrrolidine-κN)gold(I) bis(pyrrolidine-κN)gold(I) bromide (2) Data collection

**Table:**

Oxford Diffraction Xcalibur, Eos diffractometer	4461 independent reflections
Radiation source: fine-focus sealed tube	3055 reflections with *I* > 2σ(*I*)
Detector resolution: 16.1419 pixels mm^-1^	*R*_int_ = 0.143
ω scans	θ_max_ = 28.3°, θ_min_ = 2.1°
Absorption correction: multi-scan (CrysAlisPro; Agilent, 2014)	*h* = −19→19
*T*_min_ = 0.287, *T*_max_ = 0.819	*k* = −16→16
129826 measured reflections	*l* = −25→25

### Bromido(pyrrolidine-κN)gold(I) bis(pyrrolidine-κN)gold(I) bromide (2) Refinement

**Table:**

Refinement on *F*^2^	Primary atom site location: structure-invariant direct methods
Least-squares matrix: full	Secondary atom site location: difference Fourier map
*R*[*F*^2^ > 2σ(*F*^2^)] = 0.051	Hydrogen site location: mixed
*wR*(*F*^2^) = 0.130	H atoms treated by a mixture of independent and constrained refinement
*S* = 1.03	*w* = 1/[σ^2^(*F*_o_^2^) + (0.0601*P*)^2^ + 57.3562*P*] where *P* = (*F*_o_^2^ + 2*F*_c_^2^)/3
4461 reflections	(Δ/σ)_max_ = 0.001
193 parameters	Δρ_max_ = 7.82 e Å^−^^3^
90 restraints	Δρ_min_ = −2.45 e Å^−^^3^

### Bromido(pyrrolidine-κN)gold(I) bis(pyrrolidine-κN)gold(I) bromide (2) Fractional atomic coordinates and isotropic or equivalent isotropic displacement parameters (Å^2^)

**Table:**

	*x*	*y*	*z*	*U*_iso_*/*U*_eq_	Occ. (<1)
Au1	0.22755 (3)	0.62223 (3)	0.35070 (2)	0.01738 (12)	
Au2	0.24402 (3)	0.37177 (3)	0.36733 (2)	0.02106 (13)	
Br1	0.24081 (9)	0.62289 (10)	0.47285 (6)	0.0319 (3)	
Br2	0.47574 (7)	0.62096 (9)	0.29066 (7)	0.0265 (3)	
N11	0.2072 (6)	0.6225 (7)	0.2457 (5)	0.0198 (18)	
H01	0.154 (5)	0.613 (8)	0.238 (5)	0.00 (3)*	
C12	0.2422 (9)	0.7142 (9)	0.2053 (6)	0.024 (3)	
H12A	0.306033	0.728819	0.217289	0.029*	
H12B	0.206137	0.779540	0.214322	0.029*	
C13	0.2342 (13)	0.6814 (11)	0.1315 (7)	0.051 (4)	
H13A	0.285701	0.709593	0.104528	0.061*	
H13B	0.177388	0.709107	0.111426	0.061*	
C14	0.2346 (11)	0.5599 (10)	0.1315 (7)	0.042 (4)	
H14A	0.178228	0.531639	0.110969	0.051*	
H14B	0.286700	0.532125	0.105008	0.051*	
C15	0.2417 (10)	0.5284 (9)	0.2057 (7)	0.030 (4)	
H15A	0.204779	0.463819	0.215090	0.036*	
H15B	0.305281	0.512831	0.218052	0.036*	
N21	0.3785 (7)	0.3993 (7)	0.3701 (5)	0.026 (2)	
H02	0.396 (7)	0.459 (7)	0.359 (6)	0.01 (3)*	
C22	0.4148 (9)	0.3975 (12)	0.4418 (6)	0.040 (3)	
H22A	0.397647	0.330468	0.465778	0.047*	
H22B	0.392387	0.459598	0.468609	0.047*	
C23	0.5151 (9)	0.4038 (11)	0.4319 (8)	0.046 (4)	
H23A	0.547490	0.373081	0.471874	0.055*	
H23B	0.534842	0.479034	0.425349	0.055*	
C24	0.5321 (8)	0.3377 (11)	0.3674 (8)	0.046 (4)	
H24A	0.575759	0.374322	0.336788	0.056*	
H24B	0.556329	0.266040	0.379409	0.056*	
C25	0.4417 (8)	0.3276 (11)	0.3330 (7)	0.038 (3)	
H25A	0.446156	0.348998	0.284058	0.046*	
H25B	0.420235	0.252415	0.335148	0.046*	
N31	0.1091 (8)	0.3412 (8)	0.3728 (6)	0.033 (2)	
H03	0.105 (11)	0.278 (7)	0.362 (8)	0.05 (5)*	
C32	0.0779 (10)	0.3300 (13)	0.4453 (7)	0.050 (3)	
H32A	0.094769	0.259062	0.464201	0.060*	0.64 (2)
H32B	0.104374	0.386778	0.474678	0.060*	0.64 (2)
H32C	0.028784	0.276281	0.448143	0.060*	0.36 (2)
H32D	0.128372	0.305867	0.474869	0.060*	0.36 (2)
C33	−0.0217 (12)	0.341 (2)	0.4413 (11)	0.051 (6)	0.64 (2)
H33A	−0.044827	0.379924	0.482176	0.061*	0.64 (2)
H33B	−0.050730	0.269918	0.439248	0.061*	0.64 (2)
C34	−0.0420 (11)	0.404 (2)	0.3768 (13)	0.058 (8)	0.64 (2)
H34A	−0.083197	0.363328	0.346441	0.070*	0.64 (2)
H34B	−0.070657	0.473716	0.388309	0.070*	0.64 (2)
C35	0.0453 (8)	0.4212 (10)	0.3433 (7)	0.032 (3)	
H35A	0.067222	0.494943	0.352072	0.039*	0.64 (2)
H35B	0.039676	0.410829	0.293019	0.039*	0.64 (2)
H35C	−0.006657	0.384712	0.321348	0.039*	0.36 (2)
H35D	0.075975	0.466200	0.308490	0.039*	0.36 (2)
C33'	0.045 (4)	0.435 (3)	0.4683 (12)	0.072 (17)*	0.36 (2)
H33C	0.094119	0.476047	0.490651	0.087*	0.36 (2)
H33D	−0.005492	0.426470	0.501145	0.087*	0.36 (2)
C34'	0.014 (2)	0.488 (2)	0.4031 (12)	0.035 (10)*	0.36 (2)
H34C	−0.052331	0.494791	0.402725	0.042*	0.36 (2)
H34D	0.040500	0.561337	0.399837	0.042*	0.36 (2)

### Bromido(pyrrolidine-κN)gold(I) bis(pyrrolidine-κN)gold(I) bromide (2) Atomic displacement parameters (Å^2^)

**Table:**

	*U*^11^	*U*^22^	*U*^33^	*U*^12^	*U*^13^	*U*^23^
Au1	0.0260 (2)	0.01228 (19)	0.0139 (2)	0.00333 (18)	−0.00074 (14)	−0.00077 (16)
Au2	0.0371 (3)	0.0123 (2)	0.0138 (2)	0.00283 (16)	−0.00284 (15)	−0.0012 (2)
Br1	0.0612 (8)	0.0222 (6)	0.0122 (6)	0.0090 (6)	−0.0032 (5)	−0.0016 (5)
Br2	0.0183 (5)	0.0201 (5)	0.0410 (7)	0.0001 (5)	0.0013 (4)	0.0055 (5)
N11	0.018 (4)	0.017 (4)	0.024 (5)	−0.003 (4)	0.003 (4)	−0.003 (4)
C12	0.030 (8)	0.024 (6)	0.017 (8)	−0.007 (5)	−0.005 (5)	−0.001 (5)
C13	0.076 (12)	0.058 (10)	0.020 (9)	−0.018 (9)	−0.007 (8)	0.000 (8)
C14	0.062 (10)	0.047 (9)	0.018 (8)	0.020 (7)	−0.004 (7)	−0.015 (7)
C15	0.042 (9)	0.027 (6)	0.021 (9)	0.013 (5)	0.004 (5)	−0.004 (6)
N21	0.045 (6)	0.009 (4)	0.023 (6)	0.001 (4)	−0.011 (5)	0.000 (4)
C22	0.051 (9)	0.048 (8)	0.019 (7)	0.013 (7)	−0.020 (6)	−0.009 (6)
C23	0.037 (8)	0.039 (8)	0.063 (11)	0.006 (6)	−0.026 (7)	0.004 (7)
C24	0.029 (7)	0.032 (7)	0.078 (13)	0.004 (6)	−0.008 (8)	−0.014 (8)
C25	0.031 (7)	0.038 (7)	0.045 (9)	−0.006 (6)	0.008 (6)	−0.014 (6)
N31	0.047 (6)	0.024 (5)	0.027 (5)	0.007 (5)	0.006 (5)	0.001 (5)
C32	0.048 (5)	0.061 (5)	0.042 (5)	0.009 (4)	0.005 (4)	0.008 (4)
C33	0.041 (8)	0.064 (16)	0.048 (13)	0.003 (10)	0.012 (8)	0.021 (11)
C34	0.033 (8)	0.073 (18)	0.069 (16)	0.001 (10)	0.010 (9)	0.040 (14)
C35	0.037 (7)	0.028 (6)	0.033 (7)	0.001 (5)	−0.004 (5)	0.003 (5)

### Bromido(pyrrolidine-κN)gold(I) bis(pyrrolidine-κN)gold(I) bromide (2) Geometric parameters (Å, º)

**Table:**

Au1—N11	2.065 (9)	C24—H24A	0.9900
Au1—Br1	2.3837 (12)	C24—H24B	0.9900
Au1—Au2	3.1476 (6)	C25—H25A	0.9900
Au1—Au2^i^	3.1551 (6)	C25—H25B	0.9900
Au2—N21	2.021 (11)	N31—C35	1.488 (13)
Au2—N31	2.036 (12)	N31—C32	1.490 (14)
N11—C12	1.480 (12)	N31—H03	0.81 (7)
N11—C15	1.497 (12)	C32—C33'	1.460 (19)
N11—H01	0.81 (7)	C32—C33	1.483 (17)
C12—C13	1.496 (16)	C32—H32A	0.9900
C12—H12A	0.9900	C32—H32B	0.9900
C12—H12B	0.9900	C32—H32C	0.9900
C13—C14	1.514 (17)	C32—H32D	0.9900
C13—H13A	0.9900	C33—C34	1.508 (19)
C13—H13B	0.9900	C33—H33A	0.9900
C14—C15	1.500 (16)	C33—H33B	0.9900
C14—H14A	0.9900	C34—C35	1.463 (17)
C14—H14B	0.9900	C34—H34A	0.9900
C15—H15A	0.9900	C34—H34B	0.9900
C15—H15B	0.9900	C35—C34'	1.504 (18)
N21—C25	1.481 (13)	C35—H35A	0.9900
N21—C22	1.496 (12)	C35—H35B	0.9900
N21—H02	0.81 (7)	C35—H35C	0.9900
C22—C23	1.500 (15)	C35—H35D	0.9900
C22—H22A	0.9900	C33'—C34'	1.51 (2)
C22—H22B	0.9900	C33'—H33C	0.9900
C23—C24	1.523 (17)	C33'—H33D	0.9900
C23—H23A	0.9900	C34'—H34C	0.9900
C23—H23B	0.9900	C34'—H34D	0.9900
C24—C25	1.501 (15)		
			
N11—Au1—Br1	176.3 (3)	C25—C24—H24B	110.7
N11—Au1—Au2	96.6 (2)	C23—C24—H24B	110.7
Br1—Au1—Au2	83.95 (3)	H24A—C24—H24B	108.8
N11—Au1—Au2^i^	96.8 (2)	N21—C25—C24	107.2 (9)
Br1—Au1—Au2^i^	83.31 (3)	N21—C25—H25A	110.3
Au2—Au1—Au2^i^	163.074 (19)	C24—C25—H25A	110.3
N21—Au2—N31	175.4 (4)	N21—C25—H25B	110.3
N21—Au2—Au1	84.9 (2)	C24—C25—H25B	110.3
N31—Au2—Au1	96.6 (3)	H25A—C25—H25B	108.5
N21—Au2—Au1^ii^	92.2 (2)	C35—N31—C32	103.3 (9)
N31—Au2—Au1^ii^	87.2 (3)	C35—N31—Au2	118.5 (8)
Au1—Au2—Au1^ii^	167.78 (2)	C32—N31—Au2	111.8 (9)
C12—N11—C15	102.1 (8)	C35—N31—H03	119 (10)
C12—N11—Au1	118.3 (7)	C32—N31—H03	98 (10)
C15—N11—Au1	117.5 (7)	Au2—N31—H03	104 (10)
C12—N11—H01	111 (7)	C33'—C32—N31	108.0 (14)
C15—N11—H01	97 (7)	C33—C32—N31	104.4 (11)
Au1—N11—H01	109 (8)	C33—C32—H32A	110.9
N11—C12—C13	105.7 (9)	N31—C32—H32A	110.9
N11—C12—H12A	110.6	C33—C32—H32B	110.9
C13—C12—H12A	110.6	N31—C32—H32B	110.9
N11—C12—H12B	110.6	H32A—C32—H32B	108.9
C13—C12—H12B	110.6	C33'—C32—H32C	110.1
H12A—C12—H12B	108.7	N31—C32—H32C	110.1
C12—C13—C14	105.9 (11)	C33'—C32—H32D	110.1
C12—C13—H13A	110.6	N31—C32—H32D	110.1
C14—C13—H13A	110.6	H32C—C32—H32D	108.4
C12—C13—H13B	110.6	C32—C33—C34	107.0 (13)
C14—C13—H13B	110.6	C32—C33—H33A	110.3
H13A—C13—H13B	108.7	C34—C33—H33A	110.3
C15—C14—C13	105.2 (10)	C32—C33—H33B	110.3
C15—C14—H14A	110.7	C34—C33—H33B	110.3
C13—C14—H14A	110.7	H33A—C33—H33B	108.6
C15—C14—H14B	110.7	C35—C34—C33	105.6 (12)
C13—C14—H14B	110.7	C35—C34—H34A	110.6
H14A—C14—H14B	108.8	C33—C34—H34A	110.6
N11—C15—C14	105.7 (9)	C35—C34—H34B	110.6
N11—C15—H15A	110.6	C33—C34—H34B	110.6
C14—C15—H15A	110.6	H34A—C34—H34B	108.7
N11—C15—H15B	110.6	C34—C35—N31	107.0 (11)
C14—C15—H15B	110.6	N31—C35—C34'	105.6 (12)
H15A—C15—H15B	108.7	C34—C35—H35A	110.3
C25—N21—C22	102.6 (9)	N31—C35—H35A	110.3
C25—N21—Au2	120.5 (7)	C34—C35—H35B	110.3
C22—N21—Au2	112.1 (8)	N31—C35—H35B	110.3
C25—N21—H02	103 (8)	H35A—C35—H35B	108.6
C22—N21—H02	98 (8)	N31—C35—H35C	110.6
Au2—N21—H02	117 (8)	C34'—C35—H35C	110.6
N21—C22—C23	103.6 (10)	N31—C35—H35D	110.6
N21—C22—H22A	111.0	C34'—C35—H35D	110.6
C23—C22—H22A	111.0	H35C—C35—H35D	108.8
N21—C22—H22B	111.0	C32—C33'—C34'	103.8 (15)
C23—C22—H22B	111.0	C32—C33'—H33C	111.0
H22A—C22—H22B	109.0	C34'—C33'—H33C	111.0
C22—C23—C24	103.9 (10)	C32—C33'—H33D	111.0
C22—C23—H23A	111.0	C34'—C33'—H33D	111.0
C24—C23—H23A	111.0	H33C—C33'—H33D	109.0
C22—C23—H23B	111.0	C35—C34'—C33'	108.1 (14)
C24—C23—H23B	111.0	C35—C34'—H34C	110.1
H23A—C23—H23B	109.0	C33'—C34'—H34C	110.1
C25—C24—C23	105.4 (10)	C35—C34'—H34D	110.1
C25—C24—H24A	110.7	C33'—C34'—H34D	110.1
C23—C24—H24A	110.7	H34C—C34'—H34D	108.4
			
N11—Au1—Au2—N21	100.5 (4)	Au2—N21—C22—C23	172.4 (8)
Br1—Au1—Au2—N21	−83.2 (3)	N21—C22—C23—C24	−36.5 (14)
Au2^i^—Au1—Au2—N21	−41.9 (3)	C22—C23—C24—C25	17.2 (16)
N11—Au1—Au2—N31	−83.9 (4)	C22—N21—C25—C24	−31.0 (14)
Br1—Au1—Au2—N31	92.4 (3)	Au2—N21—C25—C24	−156.3 (9)
Au2^i^—Au1—Au2—N31	133.7 (3)	C23—C24—C25—N21	8.6 (16)
N11—Au1—Au2—Au1^ii^	23.8 (3)	Au1—Au2—N31—C35	22.3 (9)
Br1—Au1—Au2—Au1^ii^	−159.83 (9)	Au1^ii^—Au2—N31—C35	−146.0 (9)
Au2^i^—Au1—Au2—Au1^ii^	−118.48 (12)	Au1—Au2—N31—C32	−97.7 (8)
Au2—Au1—N11—C12	−147.6 (8)	Au1^ii^—Au2—N31—C32	94.0 (8)
Au2^i^—Au1—N11—C12	22.1 (8)	C35—N31—C32—C33'	−35 (2)
Au2—Au1—N11—C15	−24.3 (8)	Au2—N31—C32—C33'	94 (2)
Au2^i^—Au1—N11—C15	145.4 (8)	C35—N31—C32—C33	34.9 (16)
C15—N11—C12—C13	38.1 (13)	Au2—N31—C32—C33	163.4 (13)
Au1—N11—C12—C13	168.8 (9)	N31—C32—C33—C34	−24 (2)
N11—C12—C13—C14	−24.3 (17)	C32—C33—C34—C35	4 (3)
C12—C13—C14—C15	0.7 (19)	C33—C34—C35—N31	19 (3)
C12—N11—C15—C14	−37.7 (13)	C32—N31—C35—C34	−33.4 (18)
Au1—N11—C15—C14	−169.0 (9)	Au2—N31—C35—C34	−157.7 (15)
C13—C14—C15—N11	22.9 (17)	C32—N31—C35—C34'	27.0 (19)
Au1—Au2—N21—C25	−138.4 (9)	Au2—N31—C35—C34'	−97.3 (18)
Au1^ii^—Au2—N21—C25	29.7 (9)	N31—C32—C33'—C34'	28 (4)
Au1—Au2—N21—C22	100.7 (7)	N31—C35—C34'—C33'	−11 (4)
Au1^ii^—Au2—N21—C22	−91.2 (7)	C32—C33'—C34'—C35	−10 (4)
C25—N21—C22—C23	41.7 (13)		

Symmetry codes: (i) −*x*+1/2, *y*+1/2, *z*; (ii) −*x*+1/2, *y*−1/2, *z*.

### Bromido(pyrrolidine-κN)gold(I) bis(pyrrolidine-κN)gold(I) bromide (2) Hydrogen-bond geometry (Å, º)

**Table:**

*D*—H···*A*	*D*—H	H···*A*	*D*···*A*	*D*—H···*A*
N11—H01···Br2^iii^	0.81 (7)	2.70 (7)	3.498 (9)	169 (9)
N21—H02···Br2	0.81 (7)	2.69 (8)	3.477 (10)	162 (11)
C22—H22*B*···Br1	0.99	3.03	3.859 (13)	142
N31—H03···Br2^ii^	0.81 (7)	2.68 (10)	3.416 (12)	151 (14)
C32—H32*A*···Br1^ii^	0.99	2.97	3.763 (14)	138
C33—H33*A*···Br1^iv^	0.99	3.03	3.675 (18)	124

Symmetry codes: (ii) −*x*+1/2, *y*−1/2, *z*; (iii) *x*−1/2, *y*, −*z*+1/2; (iv) −*x*, −*y*+1, −*z*+1.

### Trichlorido(piperidine-κN)gold(III) (3) Crystal data

**Table:**

[AuCl_3_(C_5_H_11_N)]	*F*(000) = 712
*M**_r_* = 388.46	*D*_x_ = 2.729 Mg m^−^^3^
Monoclinic, *P*2_1_/*c*	Mo *K*α radiation, λ = 0.71073 Å
*a* = 8.47646 (16) Å	Cell parameters from 13188 reflections
*b* = 6.57436 (11) Å	θ = 2.4–30.7°
*c* = 16.9961 (3) Å	µ = 16.34 mm^−^^1^
β = 93.5133 (16)°	*T* = 100 K
*V* = 945.36 (3) Å^3^	Irregular, yellow
*Z* = 4	0.17 × 0.17 × 0.15 mm

### Trichlorido(piperidine-κN)gold(III) (3) Data collection

**Table:**

Oxford Diffraction Xcalibur, Eos diffractometer	2853 independent reflections
Radiation source: Enhance (Mo) X-ray Source	2690 reflections with *I* > 2σ(*I*)
Detector resolution: 16.1419 pixels mm^-1^	*R*_int_ = 0.037
ω scan	θ_max_ = 31.1°, θ_min_ = 2.4°
Absorption correction: multi-scan (CrysAlisPro; Agilent, 2014)	*h* = −12→12
*T*_min_ = 0.689, *T*_max_ = 1.000	*k* = −9→9
24604 measured reflections	*l* = −23→24

### Trichlorido(piperidine-κN)gold(III) (3) Refinement

**Table:**

Refinement on *F*^2^	Secondary atom site location: difference Fourier map
Least-squares matrix: full	Hydrogen site location: mixed
*R*[*F*^2^ > 2σ(*F*^2^)] = 0.017	H atoms treated by a mixture of independent and constrained refinement
*wR*(*F*^2^) = 0.030	*w* = 1/[σ^2^(*F*_o_^2^) + (0.0085*P*)^2^ + 0.3159*P*] where *P* = (*F*_o_^2^ + 2*F*_c_^2^)/3
*S* = 1.13	(Δ/σ)_max_ = 0.003
2853 reflections	Δρ_max_ = 0.92 e Å^−^^3^
96 parameters	Δρ_min_ = −0.80 e Å^−^^3^
0 restraints	Extinction correction: *SHELXL2018/3* (Sheldrick, 2015), Fc^*^=kFc[1+0.001xFc^2^λ^3^/sin(2θ)]^-1/4^
Primary atom site location: structure-invariant direct methods	Extinction coefficient: 0.00109 (7)

### Trichlorido(piperidine-κN)gold(III) (3) Special details

**Table:**

Geometry. Non-bonded contacts: 3.7100 (0.0006) Au1 - Cl2_$2 3.3365 (0.0006) Au1 - Cl3_$5 3.8344 (0.0008) Cl1 - Cl2_$6 3.8204 (0.0009) Cl1 - Cl3_$5 3.8344 (0.0008) Cl2 - Cl1_$6 3.9775 (0.0012) Cl2 - Cl2_$2 3.8204 (0.0009) Cl3 - Cl1_$1 3.8012 (0.0006) Cl3 - Cl3_$1 3.8012 (0.0006) Cl3 - Cl3_$5 112.26 ( 0.02) Au1 - Cl1 - Cl2_$6 60.17 ( 0.02) Au1 - Cl1 - Cl3_$5 152.87 ( 0.03) Au1 - Cl2 - Cl1_$6 66.44 ( 0.02) Au1 - Cl2 - Cl2_$2 109.01 ( 0.02) Au1 - Cl3 - Cl1_$1 101.40 ( 0.03) Au1 - Cl3 - Cl3_$1 60.57 ( 0.02) Au1 - Cl3 - Cl3_$5 133.79 ( 0.02) Au1 - Cl3 - Au1_$1 Operators for generating equivalent atoms: $1 -x+1, y-1/2, -z+3/2 $2 -x+1, -y+1, -z+2 $5 -x+1, y+1/2, -z+3/2 $6 -x+1, -y+2, -z+2 Dihedral angle -8.35 ( 2.02) H01 - N11 - Au1 - Cl3 Least-squares planes (x,y,z in crystal coordinates) and deviations from them (* indicates atom used to define plane) - 3.7482 (0.0032) x - 4.6421 (0.0018) y + 9.8430 (0.0029) z = 3.8214 (0.0031) * 0.0275 (0.0005) Au1 * -0.0055 (0.0007) N11 * -0.0051 (0.0006) Cl1 * -0.0085 (0.0005) Cl2 * -0.0083 (0.0005) Cl3 Rms deviation of fitted atoms = 0.0138

### Trichlorido(piperidine-κN)gold(III) (3) Fractional atomic coordinates and isotropic or equivalent isotropic displacement parameters (Å^2^)

**Table:**

	*x*	*y*	*z*	*U*_iso_*/*U*_eq_	
Au1	0.50340 (2)	0.60493 (2)	0.86802 (2)	0.00923 (4)	
Cl1	0.28810 (7)	0.81001 (9)	0.87943 (4)	0.01663 (12)	
Cl2	0.62533 (7)	0.74686 (9)	0.97772 (3)	0.01580 (12)	
Cl3	0.38860 (7)	0.46665 (9)	0.75544 (3)	0.01487 (12)	
N11	0.6977 (2)	0.4215 (3)	0.85214 (12)	0.0099 (4)	
H01	0.659 (3)	0.348 (4)	0.8139 (18)	0.023 (8)*	
C12	0.7391 (3)	0.2837 (4)	0.92065 (15)	0.0162 (5)	
H12A	0.647301	0.196029	0.930291	0.019*	
H12B	0.763017	0.366729	0.968501	0.019*	
C13	0.8809 (3)	0.1509 (4)	0.90554 (16)	0.0169 (5)	
H13A	0.851842	0.054401	0.862344	0.020*	
H13B	0.910481	0.070664	0.953489	0.020*	
C14	1.0226 (3)	0.2774 (4)	0.88339 (15)	0.0190 (6)	
H14A	1.059299	0.364332	0.928430	0.023*	
H14B	1.110477	0.186397	0.870636	0.023*	
C15	0.9751 (3)	0.4096 (4)	0.81249 (16)	0.0174 (5)	
H15A	0.946403	0.321752	0.766522	0.021*	
H15B	1.065906	0.495127	0.799304	0.021*	
C16	0.8357 (3)	0.5454 (4)	0.82901 (15)	0.0145 (5)	
H16A	0.867252	0.641410	0.872008	0.017*	
H16B	0.804704	0.625874	0.781317	0.017*	

### Trichlorido(piperidine-κN)gold(III) (3) Atomic displacement parameters (Å^2^)

**Table:**

	*U*^11^	*U*^22^	*U*^33^	*U*^12^	*U*^13^	*U*^23^
Au1	0.00840 (5)	0.00890 (5)	0.01038 (5)	0.00117 (3)	0.00037 (3)	0.00010 (3)
Cl1	0.0149 (3)	0.0182 (3)	0.0168 (3)	0.0078 (2)	0.0008 (2)	−0.0010 (2)
Cl2	0.0155 (3)	0.0165 (3)	0.0150 (3)	0.0008 (2)	−0.0019 (2)	−0.0042 (2)
Cl3	0.0127 (3)	0.0159 (3)	0.0155 (3)	0.0016 (2)	−0.0026 (2)	−0.0045 (2)
N11	0.0087 (10)	0.0085 (10)	0.0123 (10)	0.0009 (8)	−0.0009 (8)	−0.0002 (7)
C12	0.0169 (13)	0.0133 (12)	0.0186 (13)	0.0035 (10)	0.0032 (10)	0.0064 (10)
C13	0.0158 (13)	0.0142 (12)	0.0202 (13)	0.0056 (10)	−0.0026 (10)	0.0019 (10)
C14	0.0120 (13)	0.0178 (13)	0.0266 (14)	0.0045 (10)	−0.0031 (11)	−0.0023 (11)
C15	0.0098 (12)	0.0177 (13)	0.0250 (14)	0.0025 (10)	0.0039 (10)	0.0011 (10)
C16	0.0113 (12)	0.0114 (11)	0.0214 (13)	0.0012 (10)	0.0053 (10)	0.0029 (10)

### Trichlorido(piperidine-κN)gold(III) (3) Geometric parameters (Å, º)

**Table:**

Au1—N11	2.0718 (19)	C12—H12A	0.9900
Au1—Cl2	2.2757 (6)	C12—H12B	0.9900
Au1—Cl3	2.2817 (6)	C13—H13A	0.9900
Au1—Cl1	2.2868 (6)	C13—H13B	0.9900
N11—C16	1.498 (3)	C14—H14A	0.9900
N11—C12	1.500 (3)	C14—H14B	0.9900
C12—C13	1.520 (3)	C15—H15A	0.9900
C13—C14	1.527 (4)	C15—H15B	0.9900
C14—C15	1.520 (4)	C16—H16A	0.9900
C15—C16	1.520 (3)	C16—H16B	0.9900
N11—H01	0.86 (3)		
			
N11—Au1—Cl2	91.19 (6)	H12A—C12—H12B	108.0
N11—Au1—Cl3	87.73 (6)	C12—C13—H13A	109.3
Cl2—Au1—Cl3	177.91 (2)	C14—C13—H13A	109.3
N11—Au1—Cl1	177.36 (6)	C12—C13—H13B	109.3
Cl2—Au1—Cl1	90.77 (2)	C14—C13—H13B	109.3
Cl3—Au1—Cl1	90.25 (2)	H13A—C13—H13B	107.9
C16—N11—C12	112.49 (19)	C15—C14—H14A	109.8
C16—N11—Au1	110.96 (14)	C13—C14—H14A	109.8
C12—N11—Au1	113.61 (15)	C15—C14—H14B	109.8
N11—C12—C13	111.5 (2)	C13—C14—H14B	109.8
C12—C13—C14	111.8 (2)	H14A—C14—H14B	108.2
C15—C14—C13	109.4 (2)	C14—C15—H15A	109.4
C14—C15—C16	111.1 (2)	C16—C15—H15A	109.4
N11—C16—C15	110.96 (19)	C14—C15—H15B	109.4
C16—N11—H01	112 (2)	C16—C15—H15B	109.4
C12—N11—H01	108 (2)	H15A—C15—H15B	108.0
Au1—N11—H01	99 (2)	N11—C16—H16A	109.4
N11—C12—H12A	109.3	C15—C16—H16A	109.4
C13—C12—H12A	109.3	N11—C16—H16B	109.4
N11—C12—H12B	109.3	C15—C16—H16B	109.4
C13—C12—H12B	109.3	H16A—C16—H16B	108.0
			
C16—N11—C12—C13	−53.6 (3)	C13—C14—C15—C16	57.1 (3)
Au1—N11—C12—C13	179.30 (16)	C12—N11—C16—C15	54.9 (3)
N11—C12—C13—C14	54.2 (3)	Au1—N11—C16—C15	−176.54 (17)
C12—C13—C14—C15	−55.8 (3)	C14—C15—C16—N11	−57.0 (3)

### Trichlorido(piperidine-κN)gold(III) (3) Hydrogen-bond geometry (Å, º)

**Table:**

*D*—H···*A*	*D*—H	H···*A*	*D*···*A*	*D*—H···*A*
N11—H01···Cl3	0.86 (3)	2.57 (3)	3.021 (2)	114 (2)
N11—H01···Cl3^i^	0.86 (3)	2.79 (3)	3.558 (2)	150 (3)
C12—H12*B*···Cl1^ii^	0.99	2.89	3.475 (3)	119
C14—H14*B*···Cl1^iii^	0.99	2.90	3.812 (3)	154
C12—H12*A*···Cl2^ii^	0.99	2.89	3.636 (3)	133
C13—H13*A*···Cl3^i^	0.99	2.83	3.661 (3)	142
C15—H15*B*···Cl3^iv^	0.99	2.88	3.713 (3)	142
C16—H16*B*···Cl3^v^	0.99	2.82	3.607 (2)	137

Symmetry codes: (i) −*x*+1, *y*−1/2, −*z*+3/2; (ii) −*x*+1, −*y*+1, −*z*+2; (iii) *x*+1, *y*−1, *z*; (iv) *x*+1, *y*, *z*; (v) −*x*+1, *y*+1/2, −*z*+3/2.

### Bis(piperidinium) tetrachloridoaurate(III) chloride (4) Crystal data

**Table:**

(C_5_H_12_N)_2_[AuCl_4_]Cl	*D*_x_ = 1.998 Mg m^−^^3^
*M**_r_* = 546.53	Mo *K*α radiation, λ = 0.71073 Å
Orthorhombic, *I**b**a*2	Cell parameters from 2523 reflections
*a* = 19.4014 (15) Å	θ = 3.0–24.5°
*b* = 9.7612 (6) Å	µ = 8.82 mm^−^^1^
*c* = 19.1922 (11) Å	*T* = 100 K
*V* = 3634.6 (4) Å^3^	Plate, yellow
*Z* = 8	0.10 × 0.05 × 0.03 mm
*F*(000) = 2096	

### Bis(piperidinium) tetrachloridoaurate(III) chloride (4) Data collection

**Table:**

Oxford Diffraction Xcalibur, Eos diffractometer	4242 independent reflections
Radiation source: Enhance (Mo) X-ray Source	2632 reflections with *I* > 2σ(*I*)
Detector resolution: 16.1419 pixels mm^-1^	*R*_int_ = 0.075
ω scan	θ_max_ = 28.3°, θ_min_ = 2.3°
Absorption correction: multi-scan (CrysAlisPro; Agilent, 2014)	*h* = −24→25
*T*_min_ = 0.718, *T*_max_ = 1.000	*k* = −12→12
19554 measured reflections	*l* = −25→24

### Bis(piperidinium) tetrachloridoaurate(III) chloride (4) Refinement

**Table:**

Refinement on *F*^2^	Secondary atom site location: difference Fourier map
Least-squares matrix: full	Hydrogen site location: inferred from neighbouring sites
*R*[*F*^2^ > 2σ(*F*^2^)] = 0.041	H-atom parameters constrained
*wR*(*F*^2^) = 0.067	*w* = 1/[σ^2^(*F*_o_^2^) + (0.0131*P*)^2^] where *P* = (*F*_o_^2^ + 2*F*_c_^2^)/3
*S* = 1.02	(Δ/σ)_max_ < 0.001
4242 reflections	Δρ_max_ = 0.84 e Å^−^^3^
105 parameters	Δρ_min_ = −0.86 e Å^−^^3^
59 restraints	Absolute structure: Refined as an inversion twin
Primary atom site location: structure-invariant direct methods	Absolute structure parameter: 0.45 (3)

### Bis(piperidinium) tetrachloridoaurate(III) chloride (4) Special details

**Table:**

Geometry. Non-bonded contacts: 3.9947 (0.0026) Au1 - Cl1_$1 3.8135 (0.0004) Au1 - Cl6_$4 3.0850 (0.0053) Cl3 - Cl3_$5 164.57 ( 0.12) Au1 - Cl1_$1 - Au1_$1 168.05 ( 0.18) Cl3 - Cl3_$5 - Au1_$5 Operators for generating equivalent atoms: $1 -x+1/2, y-1/2, z $4 x-1/2, -y+3/2, z $5 -x, -y+1, z

### Bis(piperidinium) tetrachloridoaurate(III) chloride (4) Fractional atomic coordinates and isotropic or equivalent isotropic displacement parameters (Å^2^)

**Table:**

	*x*	*y*	*z*	*U*_iso_*/*U*_eq_	
Au1	0.15024 (2)	0.74808 (5)	0.5000 (4)	0.01752 (11)	
Cl1	0.23809 (15)	0.9043 (3)	0.5022 (7)	0.0293 (7)	
Cl2	0.1479 (5)	0.7502 (5)	0.3813 (3)	0.041 (4)	
Cl3	0.06697 (15)	0.5852 (3)	0.5004 (8)	0.0363 (8)	
Cl4	0.1459 (5)	0.7478 (6)	0.6189 (2)	0.041 (4)	
Cl5	0.500000	0.500000	0.7497 (9)	0.0209 (8)	
Cl6	0.500000	0.500000	0.4997 (9)	0.0212 (8)	
N11	0.4450 (6)	0.6793 (10)	0.6243 (7)	0.019 (3)*	
H11A	0.470289	0.655584	0.586253	0.023*	
H11B	0.463181	0.635613	0.661943	0.023*	
C12	0.3724 (9)	0.6341 (16)	0.6143 (9)	0.041 (5)*	
H12A	0.371230	0.533700	0.606908	0.049*	
H12B	0.353076	0.678608	0.572227	0.049*	
C13	0.3277 (9)	0.6715 (13)	0.6786 (9)	0.032 (5)*	
H13A	0.279477	0.641926	0.670841	0.038*	
H13B	0.345430	0.623615	0.720410	0.038*	
C14	0.3301 (7)	0.8260 (12)	0.6901 (9)	0.014 (4)*	
H14A	0.307256	0.873104	0.650700	0.017*	
H14B	0.304874	0.849496	0.733331	0.017*	
C15	0.4040 (8)	0.8740 (15)	0.6958 (8)	0.028 (4)*	
H15A	0.424040	0.837655	0.739549	0.033*	
H15B	0.404483	0.975256	0.698961	0.033*	
C16	0.4494 (7)	0.8302 (12)	0.6350 (8)	0.026 (4)*	
H16A	0.434476	0.878041	0.592151	0.031*	
H16B	0.497850	0.856231	0.644571	0.031*	
N21	0.4104 (6)	0.6232 (10)	0.3780 (7)	0.024 (3)*	
H22A	0.428402	0.583084	0.339287	0.028*	
H22B	0.422809	0.571487	0.415422	0.028*	
C22	0.3351 (7)	0.6233 (14)	0.3724 (9)	0.035 (4)*	
H23A	0.314692	0.657301	0.416377	0.042*	
H23B	0.318457	0.528585	0.364636	0.042*	
C23	0.3127 (10)	0.7123 (14)	0.3134 (9)	0.052 (6)*	
H24A	0.261699	0.715798	0.312386	0.062*	
H24B	0.328482	0.671209	0.269046	0.062*	
C24	0.3408 (8)	0.8578 (14)	0.3190 (11)	0.037 (5)*	
H25A	0.327245	0.910784	0.277207	0.045*	
H25B	0.320773	0.903412	0.360378	0.045*	
C25	0.4187 (8)	0.8560 (14)	0.3252 (9)	0.033 (4)*	
H26A	0.435924	0.949881	0.334072	0.040*	
H26B	0.439282	0.823089	0.281000	0.040*	
C26	0.4402 (8)	0.7603 (14)	0.3857 (8)	0.036 (4)*	
H21A	0.491080	0.753018	0.386922	0.043*	
H21B	0.424817	0.800577	0.430385	0.043*	

### Bis(piperidinium) tetrachloridoaurate(III) chloride (4) Atomic displacement parameters (Å^2^)

**Table:**

	*U*^11^	*U*^22^	*U*^33^	*U*^12^	*U*^13^	*U*^23^
Au1	0.01711 (19)	0.02022 (19)	0.01522 (17)	0.0008 (2)	−0.0003 (10)	0.0000 (6)
Cl1	0.0294 (18)	0.0271 (14)	0.0313 (16)	−0.0094 (12)	−0.006 (5)	0.000 (4)
Cl2	0.054 (10)	0.041 (6)	0.027 (7)	−0.015 (4)	0.005 (5)	−0.008 (3)
Cl3	0.0258 (19)	0.0326 (16)	0.0503 (18)	−0.0116 (13)	0.003 (6)	−0.012 (5)
Cl4	0.046 (9)	0.071 (8)	0.006 (5)	−0.003 (4)	0.004 (4)	0.003 (4)
Cl5	0.024 (2)	0.0228 (18)	0.0163 (17)	−0.003 (7)	0.000	0.000
Cl6	0.031 (2)	0.0233 (18)	0.0098 (16)	0.0116 (16)	0.000	0.000

### Bis(piperidinium) tetrachloridoaurate(III) chloride (4) Geometric parameters (Å, º)

**Table:**

Au1—Cl3	2.267 (3)	C13—H13A	0.9900
Au1—Cl2	2.278 (13)	C13—H13B	0.9900
Au1—Cl4	2.284 (11)	C14—H14A	0.9900
Au1—Cl1	2.287 (3)	C14—H14B	0.9900
N11—C12	1.489 (15)	C15—H15A	0.9900
N11—C16	1.490 (13)	C15—H15B	0.9900
C12—C13	1.553 (16)	C16—H16A	0.9900
C13—C14	1.525 (14)	C16—H16B	0.9900
C14—C15	1.514 (15)	N21—H22A	0.9100
C15—C16	1.523 (14)	N21—H22B	0.9100
N21—C22	1.465 (14)	C22—H23A	0.9900
N21—C26	1.466 (13)	C22—H23B	0.9900
C22—C23	1.491 (15)	C23—H24A	0.9900
C23—C24	1.526 (15)	C23—H24B	0.9900
C24—C25	1.517 (15)	C24—H25A	0.9900
C25—C26	1.547 (14)	C24—H25B	0.9900
N11—H11A	0.9100	C25—H26A	0.9900
N11—H11B	0.9100	C25—H26B	0.9900
C12—H12A	0.9900	C26—H21A	0.9900
C12—H12B	0.9900	C26—H21B	0.9900
			
Cl3—Au1—Cl2	89.8 (4)	C16—C15—H15A	108.8
Cl3—Au1—Cl4	88.2 (4)	C14—C15—H15B	108.8
Cl2—Au1—Cl4	176.70 (12)	C16—C15—H15B	108.8
Cl3—Au1—Cl1	177.0 (3)	H15A—C15—H15B	107.7
Cl2—Au1—Cl1	91.6 (3)	N11—C16—H16A	109.6
Cl4—Au1—Cl1	90.6 (4)	C15—C16—H16A	109.6
C12—N11—C16	111.5 (11)	N11—C16—H16B	109.6
N11—C12—C13	110.8 (12)	C15—C16—H16B	109.6
C14—C13—C12	109.3 (11)	H16A—C16—H16B	108.1
C15—C14—C13	110.2 (12)	C22—N21—H22A	108.8
C14—C15—C16	113.9 (11)	C26—N21—H22A	108.8
N11—C16—C15	110.5 (10)	C22—N21—H22B	108.8
C22—N21—C26	113.6 (10)	C26—N21—H22B	108.8
N21—C22—C23	110.3 (12)	H22A—N21—H22B	107.7
C22—C23—C24	112.7 (12)	N21—C22—H23A	109.6
C25—C24—C23	110.6 (12)	C23—C22—H23A	109.6
C24—C25—C26	109.5 (12)	N21—C22—H23B	109.6
N21—C26—C25	111.7 (11)	C23—C22—H23B	109.6
C12—N11—H11A	109.3	H23A—C22—H23B	108.1
C16—N11—H11A	109.3	C22—C23—H24A	109.1
C12—N11—H11B	109.3	C24—C23—H24A	109.1
C16—N11—H11B	109.3	C22—C23—H24B	109.1
H11A—N11—H11B	108.0	C24—C23—H24B	109.1
N11—C12—H12A	109.5	H24A—C23—H24B	107.8
C13—C12—H12A	109.5	C25—C24—H25A	109.5
N11—C12—H12B	109.5	C23—C24—H25A	109.5
C13—C12—H12B	109.5	C25—C24—H25B	109.5
H12A—C12—H12B	108.1	C23—C24—H25B	109.5
C14—C13—H13A	109.8	H25A—C24—H25B	108.1
C12—C13—H13A	109.8	C24—C25—H26A	109.8
C14—C13—H13B	109.8	C26—C25—H26A	109.8
C12—C13—H13B	109.8	C24—C25—H26B	109.8
H13A—C13—H13B	108.3	C26—C25—H26B	109.8
C15—C14—H14A	109.6	H26A—C25—H26B	108.2
C13—C14—H14A	109.6	N21—C26—H21A	109.3
C15—C14—H14B	109.6	C25—C26—H21A	109.3
C13—C14—H14B	109.6	N21—C26—H21B	109.3
H14A—C14—H14B	108.1	C25—C26—H21B	109.3
C14—C15—H15A	108.8	H21A—C26—H21B	107.9
			
C16—N11—C12—C13	−59.6 (17)	C26—N21—C22—C23	56.0 (16)
N11—C12—C13—C14	58.7 (17)	N21—C22—C23—C24	−55.1 (18)
C12—C13—C14—C15	−54.6 (17)	C22—C23—C24—C25	55 (2)
C13—C14—C15—C16	53.4 (16)	C23—C24—C25—C26	−53.1 (18)
C12—N11—C16—C15	55.6 (16)	C22—N21—C26—C25	−56.3 (16)
C14—C15—C16—N11	−53.2 (16)	C24—C25—C26—N21	54.1 (16)

### Bis(piperidinium) tetrachloridoaurate(III) chloride (4) Hydrogen-bond geometry (Å, º)

**Table:**

*D*—H···*A*	*D*—H	H···*A*	*D*···*A*	*D*—H···*A*
N11—H11*A*···Cl6	0.91	2.32	3.150 (16)	151
N11—H11*B*···Cl5	0.91	2.26	3.161 (16)	172
C12—H12*A*···Cl4^i^	0.99	2.82	3.788 (17)	166
C13—H13*A*···Cl4	0.99	2.96	3.78 (2)	141
C16—H16*A*···Cl3^ii^	0.99	2.68	3.601 (16)	155
N21—H22*B*···Cl6	0.91	2.31	3.150 (15)	153
N21—H22*A*···Cl5^iii^	0.91	2.35	3.246 (17)	166
C22—H23*B*···Cl2^i^	0.99	2.81	3.661 (14)	144
C23—H24*A*···Cl2	0.99	2.60	3.47 (2)	148

Symmetry codes: (i) −*x*+1/2, *y*−1/2, *z*; (ii) −*x*+1/2, *y*+1/2, *z*; (iii) *x*, −*y*+1, *z*−1/2.

### Bis(pyrrolidinium) tetrabromidoaurate(III) bromide (6) Crystal data

**Table:**

(C_4_H_10_N)_2_[AuBr_4_]Br	*D*_x_ = 2.880 Mg m^−^^3^
*M**_r_* = 740.78	Mo *K*α radiation, λ = 0.71073 Å
Orthorhombic, *I**b**a**m*	Cell parameters from 6924 reflections
*a* = 19.1275 (7) Å	θ = 2.4–30.3°
*b* = 9.4396 (13) Å	µ = 20.28 mm^−^^1^
*c* = 18.9259 (17) Å	*T* = 100 K
*V* = 3417.2 (6) Å^3^	Plate, red
*Z* = 8	0.15 × 0.12 × 0.03 mm
*F*(000) = 2688	

### Bis(pyrrolidinium) tetrabromidoaurate(III) bromide (6) Data collection

**Table:**

Oxford Diffraction Xcalibur, Eos diffractometer	2689 independent reflections
Radiation source: Enhance (Mo) X-ray Source	2205 reflections with *I* > 2σ(*I*)
Graphite monochromator	*R*_int_ = 0.085
Detector resolution: 16.1419 pixels mm^-1^	θ_max_ = 31.0°, θ_min_ = 2.1°
ω scan	*h* = −27→27
Absorption correction: multi-scan (CrysAlisPro; Agilent, 2014)	*k* = −12→13
*T*_min_ = 0.177, *T*_max_ = 1.000	*l* = −27→26
35669 measured reflections	

### Bis(pyrrolidinium) tetrabromidoaurate(III) bromide (6) Refinement

**Table:**

Refinement on *F*^2^	Primary atom site location: structure-invariant direct methods
Least-squares matrix: full	Secondary atom site location: difference Fourier map
*R*[*F*^2^ > 2σ(*F*^2^)] = 0.034	Hydrogen site location: mixed
*wR*(*F*^2^) = 0.065	H atoms treated by a mixture of independent and constrained refinement
*S* = 1.08	*w* = 1/[σ^2^(*F*_o_^2^) + (0.0204*P*)^2^ + 17.1013*P*] where *P* = (*F*_o_^2^ + 2*F*_c_^2^)/3
2689 reflections	(Δ/σ)_max_ = 0.001
88 parameters	Δρ_max_ = 2.21 e Å^−^^3^
1 restraint	Δρ_min_ = −1.24 e Å^−^^3^

### Bis(pyrrolidinium) tetrabromidoaurate(III) bromide (6) Special details

**Table:**

Geometry. Au···Br and Br···Br contacts: 3.6997 (0.0008) Au1 - Br2_$5 3.4585 (0.0003) Au1 - Br4 3.6997 (0.0008) Br2 - Au1_$3 3.3201 (0.0013) Br3 - Br3_$6 3.4585 (0.0003) Br4 - Au1_$7 171.61 ( 0.03) Au1 - Br2 - Au1_$3 149.92 ( 0.04) Au1 - Br3 - Br3_$6 Operators for generating equivalent atoms: $3 -x+1/2, y-1/2, z $4 x, y-1, z $5 -x+1/2, y+1/2, z $6 -x, -y+2, -z $7 -x, -y+1, -z $8 x, y, -z

### Bis(pyrrolidinium) tetrabromidoaurate(III) bromide (6) Fractional atomic coordinates and isotropic or equivalent isotropic displacement parameters (Å^2^)

**Table:**

	*x*	*y*	*z*	*U*_iso_*/*U*_eq_	
Au1	0.14803 (2)	0.71040 (3)	0.000000	0.01444 (7)	
Br1	0.14858 (2)	0.71441 (5)	0.12839 (3)	0.02063 (12)	
Br2	0.22029 (4)	0.49747 (8)	0.000000	0.02791 (18)	
Br3	0.07792 (3)	0.92257 (7)	0.000000	0.02033 (15)	
Br4	0.000000	0.500000	0.000000	0.01607 (19)	
Br5	0.000000	0.500000	0.250000	0.0268 (2)	
N11	0.0999 (2)	0.3577 (5)	0.1304 (3)	0.0271 (10)	
H01	0.069 (3)	0.423 (6)	0.161 (3)	0.05 (2)*	
H02	0.101 (3)	0.392 (7)	0.079 (2)	0.05 (2)*	
C12	0.0578 (3)	0.2271 (5)	0.1206 (3)	0.0299 (13)	
H12A	0.064538	0.187577	0.072666	0.036*	
H12B	0.007466	0.246555	0.127932	0.036*	
C13	0.0852 (3)	0.1262 (5)	0.1763 (3)	0.0248 (11)	
H13A	0.076595	0.026327	0.162891	0.030*	
H13B	0.063519	0.144850	0.222896	0.030*	
C14	0.1628 (3)	0.1595 (5)	0.1772 (3)	0.0280 (13)	
H14A	0.187067	0.112565	0.137389	0.034*	
H14B	0.184447	0.128375	0.222131	0.034*	
C15	0.1659 (3)	0.3205 (6)	0.1699 (4)	0.0316 (14)	
H15A	0.166824	0.366671	0.216800	0.038*	
H15B	0.207800	0.349832	0.142885	0.038*	

### Bis(pyrrolidinium) tetrabromidoaurate(III) bromide (6) Atomic displacement parameters (Å^2^)

**Table:**

	*U*^11^	*U*^22^	*U*^33^	*U*^12^	*U*^13^	*U*^23^
Au1	0.01035 (12)	0.01443 (13)	0.01854 (14)	0.00078 (9)	0.000	0.000
Br1	0.0196 (2)	0.0233 (3)	0.0190 (3)	−0.00056 (19)	−0.00249 (19)	0.0003 (2)
Br2	0.0256 (4)	0.0281 (4)	0.0300 (4)	0.0144 (3)	0.000	0.000
Br3	0.0190 (3)	0.0160 (3)	0.0260 (4)	0.0047 (2)	0.000	0.000
Br4	0.0138 (4)	0.0170 (4)	0.0174 (5)	0.0002 (3)	0.000	0.000
Br5	0.0195 (5)	0.0433 (6)	0.0177 (5)	0.000	0.000	0.000
N11	0.023 (2)	0.030 (3)	0.028 (3)	0.005 (2)	−0.002 (2)	0.007 (2)
C12	0.033 (3)	0.021 (3)	0.036 (3)	−0.002 (2)	−0.012 (3)	−0.003 (2)
C13	0.031 (3)	0.017 (2)	0.027 (3)	−0.001 (2)	−0.001 (2)	0.005 (2)
C14	0.024 (3)	0.021 (3)	0.039 (3)	0.006 (2)	0.003 (2)	0.013 (3)
C15	0.016 (2)	0.032 (3)	0.046 (4)	−0.001 (2)	−0.002 (3)	0.012 (3)

### Bis(pyrrolidinium) tetrabromidoaurate(III) bromide (6) Geometric parameters (Å, º)

**Table:**

Au1—Br3	2.4102 (7)	C12—H12B	0.9900
Au1—Br1	2.4303 (5)	C13—C14	1.517 (7)
Au1—Br1^i^	2.4303 (5)	C13—H13A	0.9900
Au1—Br2	2.4393 (8)	C13—H13B	0.9900
N11—C12	1.484 (7)	C14—C15	1.527 (7)
N11—C15	1.508 (7)	C14—H14A	0.9900
N11—H01	1.03 (4)	C14—H14B	0.9900
N11—H02	1.02 (4)	C15—H15A	0.9900
C12—C13	1.514 (7)	C15—H15B	0.9900
C12—H12A	0.9900		
			
Br3—Au1—Br1	89.399 (13)	C12—C13—C14	102.5 (4)
Br3—Au1—Br1^i^	89.399 (13)	C12—C13—H13A	111.3
Br1—Au1—Br1^i^	178.15 (3)	C14—C13—H13A	111.3
Br3—Au1—Br2	179.29 (3)	C12—C13—H13B	111.3
Br1—Au1—Br2	90.592 (13)	C14—C13—H13B	111.3
Br1^i^—Au1—Br2	90.593 (13)	H13A—C13—H13B	109.2
C12—N11—C15	108.8 (4)	C13—C14—C15	104.1 (4)
C12—N11—H01	105 (4)	C13—C14—H14A	110.9
C15—N11—H01	110 (4)	C15—C14—H14A	110.9
C12—N11—H02	99 (4)	C13—C14—H14B	110.9
C15—N11—H02	122 (4)	C15—C14—H14B	110.9
H01—N11—H02	111 (5)	H14A—C14—H14B	109.0
N11—C12—C13	104.4 (4)	N11—C15—C14	104.2 (4)
N11—C12—H12A	110.9	N11—C15—H15A	110.9
C13—C12—H12A	110.9	C14—C15—H15A	110.9
N11—C12—H12B	110.9	N11—C15—H15B	110.9
C13—C12—H12B	110.9	C14—C15—H15B	110.9
H12A—C12—H12B	108.9	H15A—C15—H15B	108.9
			
C15—N11—C12—C13	20.1 (6)	C12—N11—C15—C14	4.5 (6)
N11—C12—C13—C14	−36.7 (6)	C13—C14—C15—N11	−27.3 (6)
C12—C13—C14—C15	39.7 (6)		

Symmetry code: (i) *x*, *y*, −*z*.

### Bis(pyrrolidinium) tetrabromidoaurate(III) bromide (6) Hydrogen-bond geometry (Å, º)

**Table:**

*D*—H···*A*	*D*—H	H···*A*	*D*···*A*	*D*—H···*A*
N11—H01···Br5	1.03 (4)	2.26 (5)	3.253 (5)	161 (5)
N11—H02···Br2	1.02 (4)	2.90 (6)	3.624 (5)	128 (5)
N11—H02···Br4	1.02 (4)	2.65 (6)	3.398 (5)	130 (5)
C12—H12*B*···Br1^ii^	0.99	3.01	3.989 (6)	171
C15—H15*A*···Br1^iii^	0.99	3.05	3.847 (7)	139
C15—H15*B*···Br1^iv^	0.99	3.04	3.770 (5)	131
C15—H15*B*···Br2	0.99	3.05	3.769 (6)	130
C12—H12*A*···Br3^v^	0.99	2.87	3.691 (6)	141

Symmetry codes: (ii) −*x*, −*y*+1, *z*; (iii) *x*, −*y*+1, −*z*+1/2; (iv) −*x*+1/2, *y*−1/2, *z*; (v) *x*, *y*−1, *z*.

### Tribromido(piperidine-κN)gold(III) dichloromethane monosolvate (7) Crystal data

**Table:**

[AuBr_3_(C_5_H_11_N)]·CH_2_Cl_2_	*F*(000) = 1096
*M**_r_* = 606.77	*D*_x_ = 2.938 Mg m^−^^3^
Monoclinic, *P*2_1_/*c*	Mo *K*α radiation, λ = 0.71073 Å
*a* = 7.3473 (3) Å	Cell parameters from 8200 reflections
*b* = 22.0860 (8) Å	θ = 2.7–30.8°
*c* = 8.5066 (3) Å	µ = 19.82 mm^−^^1^
β = 96.423 (3)°	*T* = 100 K
*V* = 1371.71 (9) Å^3^	Block, red
*Z* = 4	0.15 × 0.12 × 0.10 mm

### Tribromido(piperidine-κN)gold(III) dichloromethane monosolvate (7) Data collection

**Table:**

Oxford Diffraction Xcalibur, Eos diffractometer	4139 independent reflections
Radiation source: Enhance (Mo) X-ray Source	3658 reflections with *I* > 2σ(*I*)
Detector resolution: 16.1419 pixels mm^-1^	*R*_int_ = 0.043
ω scan	θ_max_ = 30.9°, θ_min_ = 2.6°
Absorption correction: multi-scan (CrysAlisPro; Agilent, 2014)	*h* = −10→10
*T*_min_ = 0.574, *T*_max_ = 1.000	*k* = −31→31
36043 measured reflections	*l* = −12→11

### Tribromido(piperidine-κN)gold(III) dichloromethane monosolvate (7) Refinement

**Table:**

Refinement on *F*^2^	Secondary atom site location: difference Fourier map
Least-squares matrix: full	Hydrogen site location: mixed
*R*[*F*^2^ > 2σ(*F*^2^)] = 0.024	H atoms treated by a mixture of independent and constrained refinement
*wR*(*F*^2^) = 0.039	*w* = 1/[σ^2^(*F*_o_^2^) + (0.0061*P*)^2^ + 2.1369*P*] where *P* = (*F*_o_^2^ + 2*F*_c_^2^)/3
*S* = 1.15	(Δ/σ)_max_ = 0.001
4139 reflections	Δρ_max_ = 0.84 e Å^−^^3^
123 parameters	Δρ_min_ = −0.83 e Å^−^^3^
0 restraints	Extinction correction: *SHELXL2018/3* (Sheldrick 2015), Fc^*^=kFc[1+0.001xFc^2^λ^3^/sin(2θ)]^-1/4^
Primary atom site location: structure-invariant direct methods	Extinction coefficient: 0.00041 (2)

### Tribromido(piperidine-κN)gold(III) dichloromethane monosolvate (7) Special details

**Table:**

Geometry. Non-bonded contacts: 3.5658 (0.0004) Au1 - Br2_$3 3.4678 (0.0004) Au1 - Br3_$1 3.5658 (0.0004) Br2 - Au1_$1 3.3817 (0.0004) Br2 - Br3_$5 3.4678 (0.0004) Br3 - Au1_$3 3.3817 (0.0004) Br3 - Br2_$6 3.5618 (0.0021) Cl1 - Cl1_$7 88.50 ( 0.01) Au1 - Br2 - Au1_$1 158.46 ( 0.01) Au1 - Br2 - Br3_$5 90.79 ( 0.01) Au1 - Br3 - Au1_$3 155.37 ( 0.01) Au1 - Br3 - Br2_$6 146.38 ( 0.13) C1 - Cl1 - Cl1_$7Operators for generating equivalent atoms: $1 x, -y+1/2, z+1/2 $3 x, -y+1/2, z-1/2 $5 x+1, -y+1/2, z+1/2 $6 x-1, -y+1/2, z-1/2 $7 -x, -y+1, -z+1

### Tribromido(piperidine-κN)gold(III) dichloromethane monosolvate (7) Fractional atomic coordinates and isotropic or equivalent isotropic displacement parameters (Å^2^)

**Table:**

	*x*	*y*	*z*	*U*_iso_*/*U*_eq_	
Au1	0.51277 (2)	0.24454 (2)	0.25555 (2)	0.00781 (4)	
Br1	0.52418 (4)	0.35307 (2)	0.21990 (4)	0.01219 (7)	
Br2	0.79078 (4)	0.24741 (2)	0.43841 (4)	0.01246 (7)	
Br3	0.24299 (4)	0.24137 (2)	0.06300 (4)	0.01363 (7)	
N11	0.5152 (4)	0.15094 (13)	0.2928 (3)	0.0105 (6)	
H01	0.564 (5)	0.1462 (17)	0.388 (4)	0.010 (9)*	
C12	0.3327 (5)	0.12067 (16)	0.2919 (4)	0.0166 (7)	
H12A	0.262660	0.125337	0.186272	0.020*	
H12B	0.262625	0.140485	0.370457	0.020*	
C13	0.3548 (5)	0.05401 (17)	0.3310 (4)	0.0206 (8)	
H13A	0.232656	0.034650	0.324584	0.025*	
H13B	0.414508	0.049313	0.440558	0.025*	
C14	0.4697 (5)	0.02288 (17)	0.2170 (4)	0.0219 (8)	
H14A	0.488269	−0.020115	0.247579	0.026*	
H14B	0.404673	0.024218	0.108612	0.026*	
C15	0.6531 (5)	0.05400 (17)	0.2194 (4)	0.0212 (8)	
H15A	0.724300	0.034610	0.141007	0.025*	
H15B	0.722566	0.049134	0.325312	0.025*	
C16	0.6307 (5)	0.12090 (16)	0.1816 (4)	0.0158 (7)	
H16A	0.752587	0.140470	0.189969	0.019*	
H16B	0.572596	0.125882	0.071625	0.019*	
C1	0.0245 (5)	0.37793 (17)	0.2600 (4)	0.0185 (7)	
H1A	0.145117	0.357439	0.273057	0.022*	
H1B	−0.071860	0.346408	0.252639	0.022*	
Cl1	0.00338 (15)	0.42462 (5)	0.42603 (12)	0.0294 (2)	
Cl2	0.00380 (16)	0.42122 (5)	0.08506 (12)	0.0327 (2)	

### Tribromido(piperidine-κN)gold(III) dichloromethane monosolvate (7) Atomic displacement parameters (Å^2^)

**Table:**

	*U*^11^	*U*^22^	*U*^33^	*U*^12^	*U*^13^	*U*^23^
Au1	0.00905 (6)	0.00586 (6)	0.00833 (6)	−0.00023 (5)	0.00018 (4)	0.00005 (5)
Br1	0.01637 (16)	0.00648 (16)	0.01348 (16)	0.00051 (12)	0.00062 (12)	0.00047 (12)
Br2	0.01135 (14)	0.01168 (17)	0.01346 (15)	−0.00178 (12)	−0.00249 (11)	0.00151 (12)
Br3	0.01223 (15)	0.01432 (18)	0.01331 (15)	−0.00090 (13)	−0.00310 (12)	0.00090 (13)
N11	0.0165 (14)	0.0063 (14)	0.0080 (13)	0.0013 (11)	−0.0018 (11)	−0.0003 (10)
C12	0.0186 (18)	0.0109 (18)	0.0216 (18)	−0.0050 (14)	0.0082 (14)	−0.0023 (14)
C13	0.032 (2)	0.0105 (19)	0.0203 (18)	−0.0055 (15)	0.0068 (16)	−0.0003 (15)
C14	0.038 (2)	0.0085 (18)	0.0198 (19)	0.0001 (16)	0.0037 (17)	−0.0009 (14)
C15	0.031 (2)	0.0091 (19)	0.0233 (19)	0.0028 (15)	0.0042 (16)	−0.0017 (15)
C16	0.0212 (18)	0.0104 (18)	0.0162 (17)	0.0036 (14)	0.0038 (14)	0.0002 (14)
C1	0.0178 (18)	0.0147 (18)	0.0239 (19)	0.0012 (14)	0.0061 (15)	0.0030 (15)
Cl1	0.0363 (6)	0.0285 (6)	0.0232 (5)	0.0024 (4)	0.0023 (4)	−0.0021 (4)
Cl2	0.0477 (7)	0.0282 (6)	0.0239 (5)	0.0064 (5)	0.0118 (5)	0.0081 (4)

### Tribromido(piperidine-κN)gold(III) dichloromethane monosolvate (7) Geometric parameters (Å, º)

**Table:**

Au1—N11	2.091 (3)	C12—H12A	0.9900
Au1—Br1	2.4187 (4)	C12—H12B	0.9900
Au1—Br2	2.4260 (3)	C13—H13A	0.9900
Au1—Br3	2.4270 (3)	C13—H13B	0.9900
N11—C16	1.495 (4)	C14—H14A	0.9900
N11—C12	1.497 (4)	C14—H14B	0.9900
C12—C13	1.514 (5)	C15—H15A	0.9900
C13—C14	1.520 (5)	C15—H15B	0.9900
C14—C15	1.511 (5)	C16—H16A	0.9900
C15—C16	1.517 (5)	C16—H16B	0.9900
C1—Cl2	1.761 (4)	C1—H1A	0.9900
C1—Cl1	1.769 (4)	C1—H1B	0.9900
N11—H01	0.85 (4)		
			
N11—Au1—Br1	177.24 (8)	C14—C13—H13A	109.5
N11—Au1—Br2	86.35 (8)	C12—C13—H13B	109.5
Br1—Au1—Br2	90.913 (12)	C14—C13—H13B	109.5
N11—Au1—Br3	93.80 (8)	H13A—C13—H13B	108.1
Br1—Au1—Br3	88.920 (12)	C15—C14—H14A	109.6
Br2—Au1—Br3	177.454 (13)	C13—C14—H14A	109.6
C16—N11—C12	111.7 (3)	C15—C14—H14B	109.6
C16—N11—Au1	109.8 (2)	C13—C14—H14B	109.6
C12—N11—Au1	116.6 (2)	H14A—C14—H14B	108.2
N11—C12—C13	111.1 (3)	C14—C15—H15A	109.4
C12—C13—C14	110.7 (3)	C16—C15—H15A	109.4
C15—C14—C13	110.1 (3)	C14—C15—H15B	109.4
C14—C15—C16	111.4 (3)	C16—C15—H15B	109.4
N11—C16—C15	110.7 (3)	H15A—C15—H15B	108.0
Cl2—C1—Cl1	110.5 (2)	N11—C16—H16A	109.5
C16—N11—H01	110 (2)	C15—C16—H16A	109.5
C12—N11—H01	104 (2)	N11—C16—H16B	109.5
Au1—N11—H01	105 (3)	C15—C16—H16B	109.5
N11—C12—H12A	109.4	H16A—C16—H16B	108.1
C13—C12—H12A	109.4	Cl2—C1—H1A	109.5
N11—C12—H12B	109.4	Cl1—C1—H1A	109.5
C13—C12—H12B	109.4	Cl2—C1—H1B	109.5
H12A—C12—H12B	108.0	Cl1—C1—H1B	109.5
C12—C13—H13A	109.5	H1A—C1—H1B	108.1
			
C16—N11—C12—C13	56.4 (4)	C13—C14—C15—C16	−56.3 (4)
Au1—N11—C12—C13	−176.2 (2)	C12—N11—C16—C15	−55.8 (4)
N11—C12—C13—C14	−56.4 (4)	Au1—N11—C16—C15	173.2 (2)
C12—C13—C14—C15	56.3 (4)	C14—C15—C16—N11	56.0 (4)

### Tribromido(piperidine-κN)gold(III) dichloromethane monosolvate (7) Hydrogen-bond geometry (Å, º)

**Table:**

*D*—H···*A*	*D*—H	H···*A*	*D*···*A*	*D*—H···*A*
N11—H01···Br1^i^	0.85 (4)	2.87 (4)	3.627 (3)	148 (3)
C14—H14*A*···Br1^ii^	0.99	2.82	3.788 (4)	167
C16—H16*B*···Br1^iii^	0.99	3.01	3.960 (3)	161
C1—H1*B*···Br1^iv^	0.99	2.95	3.695 (4)	133
C1—H1*B*···Br2^iv^	0.99	2.94	3.761 (4)	141
C12—H12*A*···Cl1^iii^	0.99	2.97	3.855 (4)	150
C15—H15*B*···Cl2^v^	0.99	2.93	3.849 (4)	156
C1—H1*A*···Br1	0.99	2.87	3.765 (4)	150

Symmetry codes: (i) *x*, −*y*+1/2, *z*+1/2; (ii) −*x*+1, *y*−1/2, −*z*+1/2; (iii) *x*, −*y*+1/2, *z*−1/2; (iv) *x*−1, *y*, *z*; (v) *x*+1, −*y*+1/2, *z*+1/2.

## References

[bb1] Agilent (2014). *CrysAlis PRO*. Agilent Technologies, now Rigaku Oxford Diffraction, Yarnton, England.

[bb2] Ahrens, B., Friedrichs, S., Herbst-Irmer, R. & Jones, P. G. (2000). *Eur. J. Inorg. Chem.* pp. 2017–2029.

[bb3] Ahrens, B., Friedrichs, S., Herbst-Irmer, R. & Jones, P. G. (2003). *CSD Communication* (CCDC-204636). CCDC, Cambridge, England. https://dx.doi.org/10.5571/cc6vy54.

[bb4] Ahrens, B., Jones, P. G. & Fischer, A. K. (1999). *Eur. J. Inorg. Chem.* pp. 1103–1110.

[bb5] Brammer, L. (2003). *Dalton Trans.* pp. 3145–3157.

[bb6] Bruno, I. J., Cole, J. C., Edgington, P. R., Kessler, M., Macrae, C. F., McCabe, P., Pearson, J. & Taylor, R. (2002). *Acta Cryst.* B**58**, 389–397. 10.1107/s0108768102003324 12037360

[bb7] Döring, C. & Jones, P. G. (2013). *Z. Naturforsch. B*, **68**, 474–492.

[bb8] Döring, C. & Jones, P. G. (2016). *Z. Anorg. Allg. Chem.* **642**, 930–936.

[bb9] Döring, C. & Jones, P. G. (2018*a*). *Z. Naturforsch. B*, **73**, 43–74.

[bb10] Döring, C. & Jones, P. G. (2018*b*). *Z. Naturforsch. B*, **73**, 975–978.

[bb11] Elgemeie, G. E. H., Hanfy, N., Hopf, H. & Jones, P. G. (1998). *Acta Cryst.* C**54**, 136–138.

[bb12] Elgemeie, G. H., Sayed, S. H. & Jones, P. G. (2013). *Acta Cryst.* C**69**, 90–92. 10.1107/S0108270112049906 23282923

[bb13] Groom, C. R., Bruno, I. J., Lightfoot, M. P. & Ward, S. C. (2016). *Acta Cryst.* B**72**, 171–179. 10.1107/S2052520616003954 PMC4822653 27048719

[bb14] Guy, J. J., Jones, P. G., Mays, M. J. & Sheldrick, G. M. (1977). *J. Chem. Soc. Dalton Trans.* pp. 8–10.

[bb15] Jones, P. G. (1981). *Gold Bull.* **14**, 102–118.

[bb16] Jones, P. G. & Ahrens, B. (1997). *Chem. Ber.* **130**, 1813–1814.

[bb17] Metrangolo, P., Meyer, F., Pilati, T., Resnati, G. & Terraneo, G. (2008). *Angew. Chem. Int. Ed.* **47**, 6114–6127. 10.1002/anie.200800128 18651626

[bb18] Pearson, R. G. (1963). *J. Am. Chem. Soc.* **85**, 3533–3539.

[bb19] Schmidbaur, H. & Schier, A. (2008). *Chem. Soc. Rev.* **37**, 1931–1951. 10.1039/b708845k 18762840

[bb20] Schmidbaur, H. & Schier, A. (2012). *Chem. Soc. Rev.* **41**, 370–412. 10.1039/c1cs15182g 21863191

[bb21] Sheldrick, G. M. (2008). *Acta Cryst.* A**64**, 112–122. 10.1107/S0108767307043930 18156677

[bb22] Sheldrick, G. M. (2015). *Acta Cryst.* C**71**, 3–8.

[bb23] Siemens (1994). *XP*. Siemens Analytical X–Ray Instruments, Madison, Wisconsin, USA.

[bb24] Strey, M., Döring, C. & Jones, P. G. (2018). *Z. Naturforsch. B*, **73**, 125–147.

[bb25] Teci, M., Hueber, D., Pale, P., Toupet, L., Blanc, A., Brenner, E. & Matt, D. (2017). *Chem. Eur. J.* **23**, 7809–7818. 10.1002/chem.201701129 28523648

[bb26] Tomás-Mendivil, E., Toullec, P. Y., Borge, J., Conejero, S., Michelet, V. & Cadierno, V. (2013). *ACS Catal.* **3**, 3086–3098.

[bb27] Upmann, D., Koneczny, M., Rass, J. & Jones, P. G. (2019). *Z. Naturforsch. B*, **74**, 389–404.

[bb28] Upmann, D., Näther, C., Jess, I. & Jones, P. G. (2017). *Z. Anorg. Allg. Chem.* **643**, 311–316.

